# History-dependent physiological adaptation to lethal genetic modification under antibiotic exposure

**DOI:** 10.7554/eLife.74486

**Published:** 2022-05-10

**Authors:** Yuta Koganezawa, Miki Umetani, Moritoshi Sato, Yuichi Wakamoto

**Affiliations:** 1 Department of Basic Science, Graduate School of Arts and Sciences, The University of Tokyo Meguro-ku Japan; 2 Research Center for Complex Systems Biology, The University of Tokyo Tokyo Japan; 3 Department of Life Sciences, Graduate School of Arts and Sciences, The University of Tokyo Tokyo Japan; 4 Universal Biology Institute, The University of Tokyo Tokyo Japan; Harvard Medical School United States; Weizmann Institute of Science Israel

**Keywords:** adaptation, antibiotic resistance, single-cell analysis, optogenetics, homeostasis, ribosome, *E. coli*

## Abstract

Genetic modifications, such as gene deletion and mutations, could lead to significant changes in physiological states or even cell death. Bacterial cells can adapt to diverse external stresses, such as antibiotic exposure, but can they also adapt to detrimental genetic modification? To address this issue, we visualized the response of individual *Escherichia coli* cells to deletion of the antibiotic resistance gene under chloramphenicol (Cp) exposure, combining the light-inducible genetic recombination and microfluidic long-term single-cell tracking. We found that a significant fraction (∼40%) of resistance-gene-deleted cells demonstrated a gradual restoration of growth and stably proliferated under continuous Cp exposure without the resistance gene. Such physiological adaptation to genetic modification was not observed when the deletion was introduced in 10 hr or more advance before Cp exposure. Resistance gene deletion under Cp exposure disrupted the stoichiometric balance of ribosomal large and small subunit proteins (RplS and RpsB). However, the balance was gradually recovered in the cell lineages with restored growth. These results demonstrate that bacterial cells can adapt even to lethal genetic modifications by plastically gaining physiological resistance. However, the access to the resistance states is limited by the environmental histories and the timings of genetic modification.

## Introduction

Bacteria in nature are constantly challenged by environmental and genetic perturbations and must be robust against them for survival. Bacterial cells possess programs to adapt or resist such perturbations. For example, under the conditions of nutrient deprivation, *E. coli* and related bacteria provoke RpoS-mediated general stress response and globally change the metabolic and gene expression profiles to protect themselves from the stress (Battesti et al., 2011). DNA damage also induces SOS response, which promotes both DNA repair and mutagenesis (Walker, 1996; Taddei et al., 1995). If the mutations in essential genetic elements remain unrepaired, their influence will be propagated to cellular phenotypes or even cause cell death. How rapidly new genetic changes alter cellular phenotypes and whether they always give rise to the same phenotypes in given environments are fundamental questions in genetics (Ryan, 1955; Sun et al., 2018). Nevertheless, such time-dependent and redundant genotype-phenotype correspondences are usually deemed negligible or insignificant in most genetics analyses.

However, the evidence suggests that biological systems can buffer or compensate for the impact of genetic changes at the cytoplasmic and physiological levels (Waddington, 1942). For example, specific molecular chaperons could hinder genetic variations from manifesting as morphological and growth phenotypes in fruit flies (Rutherford and Lindquist, 1998), plants (Queitsch et al., 2002), and bacteria (Fares et al., 2002). Furthermore, the loss of biological functions caused by genetic changes can be compensated for by modulating the RNA and protein levels of the mutated or other related genes in many organisms (El-Brolosy and Stainier, 2017). In *Bacillus subtilis*, the expression noise of mutant sporulation regulator results in the partial penetrance of its influence to spore-forming phenotypes (Eldar et al., 2009). These observations across diverse organisms suggest that phenotypic consequences of genetic modifications can be modulated based on environmental and physiological contexts, which may promote the survival and evolution of the organisms.

Despite these experimental implications, it remains elusive whether bacterial cells can circumvent even lethal genetic modifications such as antibiotic resistance gene deletion under antibiotic exposure. Furthermore, how cells initially respond to the genetic changes and how their physiological and phenotypic states are modulated in longer timescales are poorly characterized. However, addressing these issues requires precisely defining the timings of genetic changes and tracking individual cells for a long period to unravel their phenotypic transitions and consequences (Sun et al., 2018).

In this study, we resolve these technical issues by combining the photoactivatable Cre (PA-Cre) light-inducible genetic recombination technique (Kawano et al., 2016) and microfluidic long-term single-cell tracking (Wang et al., 2010). We induced a pre-designed deletion of chromosomally encoded and fluorescently tagged drug resistance gene in *E. coli* directly in the microfluidic device. The results show that all of the resistance-gene-deleted cells under continuous drug exposure showed a decline in growth in 5–7 generations, but a fraction of the resistance-gene-deleted cells gradually restored their growth without additional mutations. In contrast, no cells restored growth when the same deletion was introduced 10 hr or more in advance before drug exposure. Therefore, bacterial cells can physiologically adapt to lethal genetic modifications. However, its feasibility depends on environmental histories, the timings of genetic modifications, and the severity of the antibiotic stress.

## Results

### Resistance gene deletion in *E. coli* by blue-light illumination

**Figure 1.**
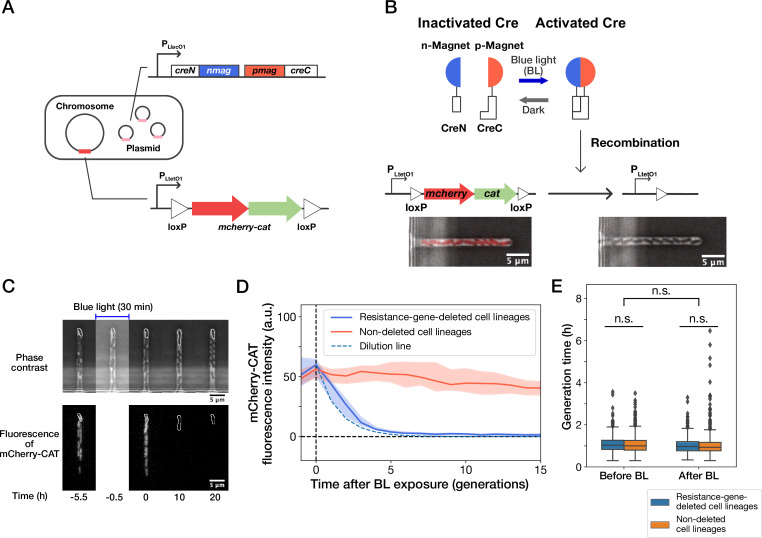
Live-cell monitoring of phenotypic transitions in response to gene deletion. (**A**) Schematic drawing of an *E. coli* strain, YK0083. This strain harbors the photo-removable *mcherry-cat* gene on the chromosome and a low-copy plasmid carrying the *pa-cre* genes (*creN-nmag* and *pmag-creC*). (**B**) The PA-Cre system. Blue-light illumination provokes the dimerization of two PA-Cre fragments and induces the deletion of the *mcherry-cat* gene. The micrographs represent the combined images of phase contrast and mCherry-CAT fluorescence channels. Left and right images show the cells before and after blue-light illumination, respectively. (**C**) Representative time-lapse images of gene-deletion experiments. The upper and lower images show the cells in phase contrast and mCherry-CAT fluorescence channels, respectively. The cell lineages at the closed end of the growth channel (outlined in white) were monitored. A 30-min blue-light illumination starting at t = –0.5 hr led to the loss of mCherry-CAT fluorescence signals in this cell lineage. (**D**) The transitions of mCherry-CAT fluorescence intensities in resistance-gene-deleted (blue) and non-deleted (red) cell lineages. Lines and shaded areas represent the medians and the 25–75% ranges, respectively. The cyan broken line represents the expected fluorescence decay curve when the fluorescence intensity decreases to half in each generation. (**E**) Generation time of resistance-gene-deleted and non-deleted cells 10 generations before and after blue-light illumination. The middle line and both edges of the boxes represent the medians and the 25–75% ranges of generation time. Whiskers indicate the minimum and maximum of the data except for the outliers. The points represent the outliers. No significant differences in generation time were detected between the groups at the significance level of 0.01 (p = 0.47 for before BL, p = 0.027 for after BL, p = 0.055 for before BL vs after BL, Mann-Whitney U test).

To investigate the response of individual cells to antibiotic resistance gene deletion, we constructed an *E. coli* strain expressing chloramphenicol acetyltransferase (CAT) tagged with mCherry red fluorescent protein (Figure 1A). CAT confers resistance to chloramphenicol (Cp) by acetylating Cp (Shaw, 1967). The *mcherry-cat* resistance gene was integrated on the chromosome along with the upstream and downstream *loxP* sequences so that the resistance gene could be excised by Cre recombinase (Figure 1A). We also introduced the PA-Cre recombination system, which consists of split Cre-recombinase fragments, called CreC and CreN, attached to p-Magnet (p-Mag) and n-Magnet (n-Mag) monomers, respectively (Figure 1B; Kawano et al., 2015). p-Mag and n-Mag are heterodimers derived from the fungal photo-receptor, Vivid (Kawano et al., 2015), which heterodimerize upon blue-light illumination. In the PA-Cre system, blue-light illumination leads to heterodimerizations of p-Mag-CreC and n-Mag-CreN fragments and recovers Cre recombination activity (Figure 1B; Kawano et al., 2016). Therefore, this system allows us to induce the resistance gene deletion at arbitrary timings by blue-light illumination. The original PA-Cre system was designed for use in mammalian cells (Kawano et al., 2016); we thus replaced the plasmid backbone and the promoter for use in *E. coli*.

**Figure 1—figure supplement 1.**
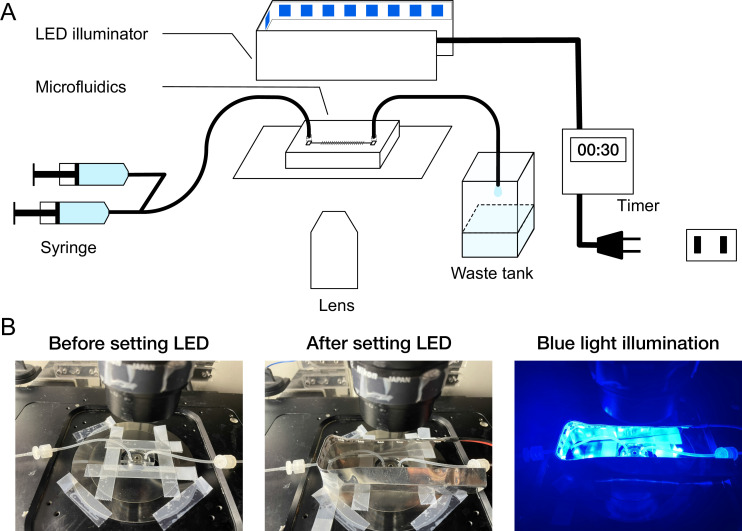
On-stage blue-light LED illuminator. (**A**) Schematic diagram of microscope setup. A custom blue-light LED illuminator was placed on the microscope stage to illuminate the microfluidic device. The timing and duration of illumination were controlled by an external timer. (**B**) Photo images of the microfluidic device and LED illuminator mounted on the microscope stage.

**Figure 1—figure supplement 2.**
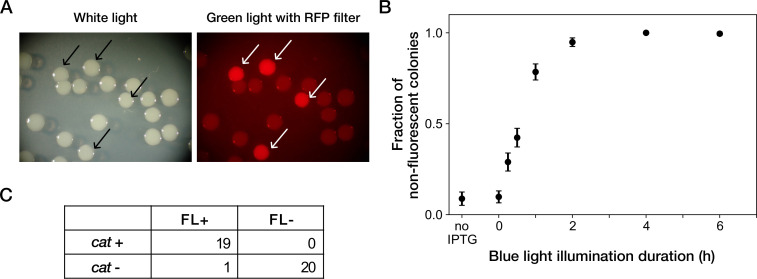
Resistance gene deletion by blue-light illumination in batch cultures. (**A**) Colonies of YK0083 cells inoculated after blue-light illumination. The YK0083 cells were exposed to blue light for 1 hr in batch culture and spread on agar. The colonies were photographed under the exposure of ambient light (left) and excitation light (right). The colonies of the cells retaining the *mcherry-cat* gene were visually detectable under the excitation light (arrows). (**B**) Relationship between blue-light illumination duration and fractions of non-fluorescent colonies. Black points represent the means. Error bars represent standard errors (N = 239 for no IPTG; N = 327 for 0 hr; N = 335 for 0.25 hr; N = 373 for 0.5 hr; N = 344 for 1 hr; N = 350 for 2 hr; N = 296 for 4 hr; N = 209 for 6 hr). ‘no IPTG’ represents the condition where the cells were cultured in the M9 medium without IPTG and not exposed to blue light. (**C**) The correspondence between mCherry-CAT fluorescence and presence of the *cat* resistance gene. FL +and FL- represent the presence/absence of mCherry fluorescence visually inspected for the colonies. *cat* +and *cat*- represent the presence/absence of the *cat* gene detected by colony PCR. All non-fluorescent colonies had lost the *cat* gene. All but one fluorescent colony retained the *cat* gene.

We first analyzed the response of the constructed strain YK0083 to blue-light illumination under drug-free conditions. Single-cell observations with the mother machine microfluidic device (Wang et al., 2010) and custom stage-top LED illuminator (Figure 1—figure supplement 1) revealed that 30-min blue-light exposure (λ= 464∼474 nm, 6.8 mW at the specimen position) led to the loss of mCherry-CAT fluorescence in 25% (50/200) cells (Figure 1C and D, and Video 1), suggesting the deletion of the *mcherry-cat* gene in these cell lineages. The fluorescence signals decayed to the background level in 4–5 generations, and the decay kinetics was consistent with the dilution by growth (Figure 1D). Furthermore, the 30 min blue-light illumination and genetic recombination did not affect the growth of individual cells (Figure 1E).

**Video 1.** Deletion of *mcherry-cat* gene by blue-light illumination under drug-free conditions. YK0083 cells were cultured in the mother machine flowing the M9 minimal medium and exposed to blue light from t = –30 min to 0 min (marked with ‘ + BL’). The merged images of phase contrast (grayscale) and mCherry-CAT fluorescence (red) channels are shown. The fluorescence of mCherry-CAT was gradually lost after blue-light illumination in this cell lineage. Scale bar, 5 µm.

Extending illumination duration increased the frequency of cells showing loss of fluorescence, reaching 100% (n=296) in 4 hr (Figure 1—figure supplement 2A and Figure 1—figure supplement 2B). We confirmed the correspondence between the loss of mCherry fluorescence and *cat* gene deletion by colony PCR (Figure 1—figure supplement 2C).

### Fractional continuation of growth against resistance gene deletion under drug exposure

**Figure 2.**
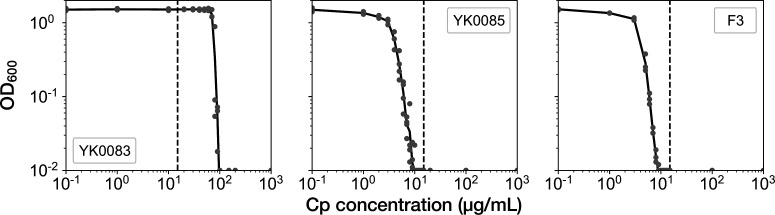
MIC tests. Gray points represent the OD_600_ of the indicated strains after a 23 hr incubation period in the M9 media containing the corresponding concentrations of Cp. The minimum concentration where OD_600_ became lower than 0.01 was adopted as the MIC for each strain. A total of 15 µg/mL of Cp was used in the time-lapse experiments (dashed line). The measurements were repeated at least thrice.

We next induced the deletion of the resistance gene in YK0083 cells in the mother machine, continuously flowing a medium containing 15 µg/mL of Cp. This drug concentration was 1.5-fold higher than the minimum inhibitory concentration (MIC) of the non-resistant strain YK0085 (10 µg/mL), which was constructed by illuminating the YK0083 cells by blue light in batch culture (Figure 2). Therefore, it was expected that this drug concentration would inhibit the growth of resistance-gene-deleted cells. This Cp concentration was significantly lower than the MIC of resistant YK0083 cells (100 µg/mL, Figure 2) and did not influence their elongation rates (Figure 2—figure supplement 1).

**Figure 2—figure supplement 1.**
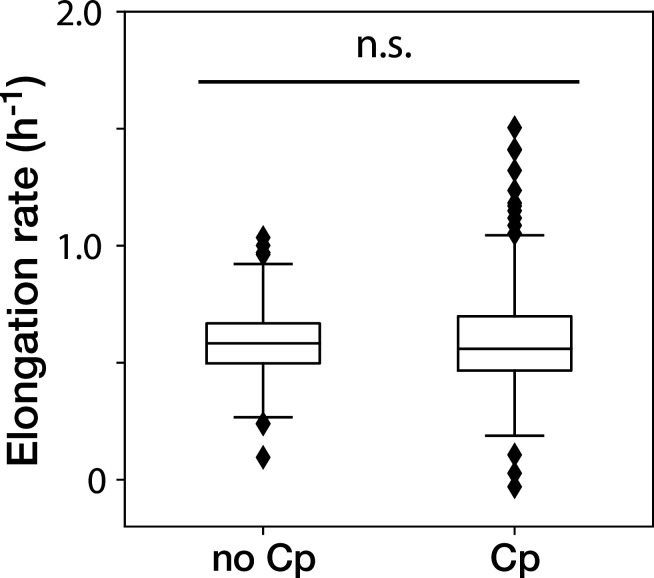
The Cp concentration used in the time-lapse measurements caused no significant effect on the growth of non-deleted YK0083 cells. Box plots show the elongation rates of the YK0083 cells growing in the mother machine. ‘no Cp’ denotes the drug-free condition; ‘Cp’ denotes the condition under the exposure of 15 µg/mL of Cp. The elongation rates were obtained from the non-deleted cell lineages up to 10 generations after blue-light illumination. We detected no significant differences in elongation rates between the two conditions (p = 0.63, Welch’s t-test).

**Figure 3.**
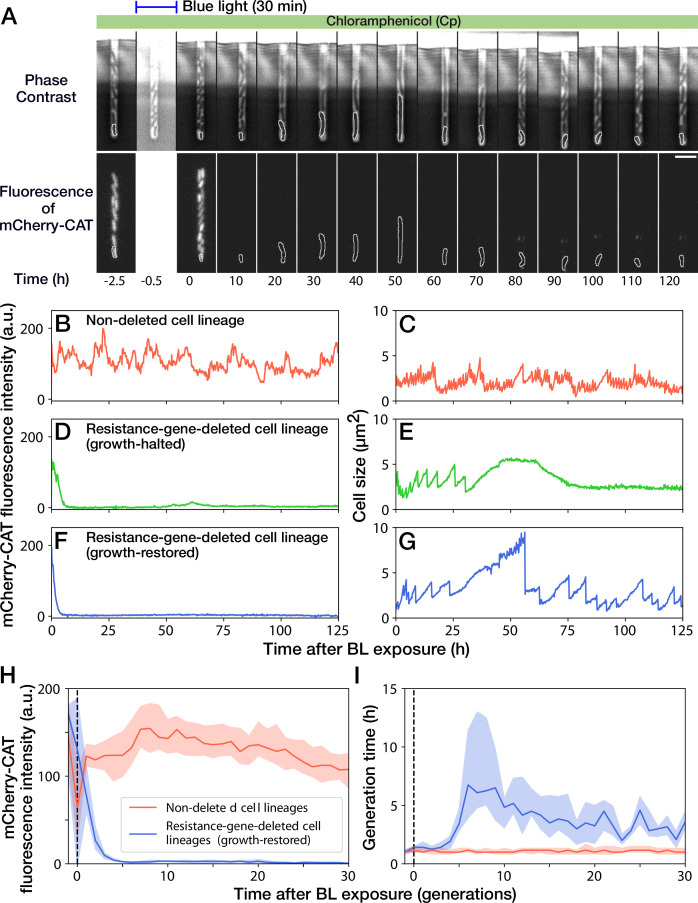
Growth continuation under Cp exposure against *cat* gene deletion. (**A**) Time-lapse images of a cell lineage that continued growth and division against *mcherry-cat* gene deletion. The upper and lower sequences show phase contrast and mCherry fluorescence images, respectively. Blue light was illuminated from t = -0.5 h to 0 hr. The cells at the closed end of the growth channel were monitored during the experiment, which are outlined in white on the images. Scale bar, 5 µm. (**B–G**) The transitions of mCherry-CAT fluorescence intensities and cell size in single-cell lineages. (**B, C**) Non-deleted cell lineage. (**D, E**) Growth-halted resistance-gene-deleted cell lineage. The decrease in cell size after 60 hr was due to the shrinkage of the cell body. (**F, G**) Growth-restored resistance-gene-deleted cell lineage. (**H, I**) The transitions of mCherry-CAT fluorescence intensities (**H**) and generation time (**I**). The lines and shaded areas represent the medians and the 25–75% ranges, respectively. Red represents non-deleted cell lineages. Blue represents growth-restored resistance-gene-deleted cell lineages. The transitions are shown in generations.

A 30-min blue-light illumination induced the *mcherry-cat* gene deletion in 24.5% (343/1399) in YK0083 cells (Figure 3A and Video 2). While non-deleted cells continued to grow with their generation time (interdivision time) and mCherry-CAT fluorescence intensity nearly unaffected (Figure 3B and C), resistance-gene-deleted cells gradually showed a decline in growth (Figure 3D–G). The fluorescence intensity of the resistance-gene-deleted cells decayed to the background levels in 4–5 generations (Figure 3D, F and H), and their generation time also increased correspondingly (Figure 3E, G and I). However, while the growth of 62.7% (163/260) of resistance-gene-deleted cells was eventually stopped, the other 37.3% cells restored and continued their growth over 30 generations without the *cat* resistance gene (Figure 3H and I). The generation time of these cell lineages recovered from 6.3 hr (the median of 6th-9th generations) to 3.0 hr (the median of 21st-30th generations) under the continuous drug exposure (Figure 3I). The elongation rate transitions across multiple generations also manifested the growth recovery (Figure 3—figure supplement 1).

**Figure 3—figure supplement 1.**
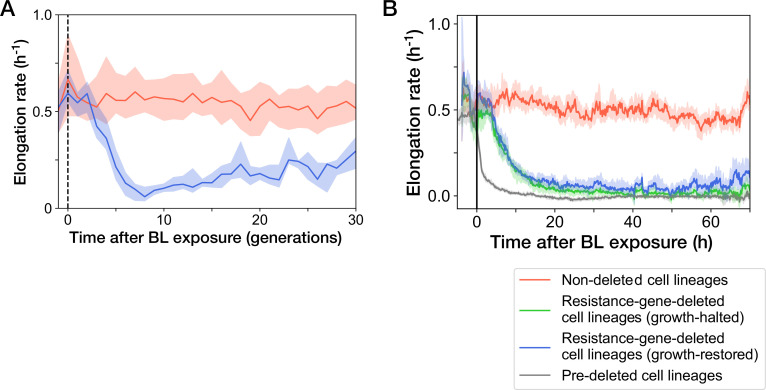
Transitions of elongation rates after blue-light illumination. The YK0083 cells were exposed to blue light for 30 min from t = -0.5 hr to 0 hr. (**A**) Transitions of elongation rates of resistance-gene-deleted YK0083 cell lineages in generations. The lines and shaded areas represent the medians and the 25–75% data ranges, respectively. (**B**) Transitions of elongation rates in hours after blue-light illumination. The lines represent the medians of elongation rate at each time point. The shaded areas show the 95% error ranges of the median estimated by resampling the cell lineages 1000 times. Red represents the non-deleted cell lineages. Blue represents the growth-restored resistance-gene-deleted cell lineages. Green represents the growth-halted resistance-gene-deleted cell lineages. Gray represents the pre-deleted YK0085 cell lineages.

**Figure 3—figure supplement 2.**
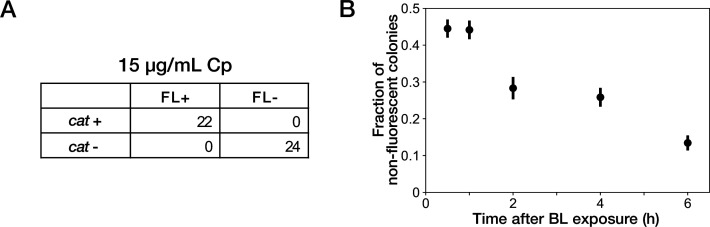
Resistance gene deletion in batch cultures under Cp exposure. (**A**) The correspondence between mCherry-CAT fluorescence and presence of the *cat* resistance gene. Deletions of resistance gene were induced under exposure to 15 µg/mL of Cp. FL +and FL- represent the presence/absence of mCherry fluorescence visually inspected for the colonies. *cat* +and *cat*- represent the presence/absence of the *cat* gene detected by colony PCR. All non-fluorescent colonies had lost the *cat* gene. All fluorescent colonies retained the *cat* gene. (**B**) Competition between resistance-gene-deleted and non-deleted cells under Cp exposure. Batch cultures of YK0083 cells were illuminated by blue light for 30 min, and the fractions of resistance-gene-deleted cells were quantified by the proportions of non-fluorescence colonies in the cultures sampled at several time points after blue-light illumination. Black points represent the means. Error bars represent standard errors (N = 409 for 0.5 hr; N = 385 for 1 hr; N = 219 for 2 hr; N = 298 for 4 hr; N = 283 for 6 hr).

**Figure 3—figure supplement 3.**
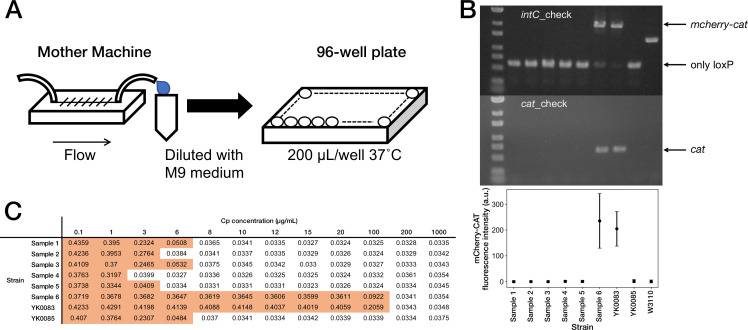
Correspondence between fluorescence loss and *mcherry-cat* gene deletion in the cells sampled from the mother machine. (**A**) The scheme for acquiring the resistance-gene-deleted cells from the mother machine. The culture media flowing out from the mother machine were sampled to obtain the cells that lost the *mcherry-cat* gene. The media were diluted serially, and the limited-diluted media were taken in 96-well plates and incubated. (**B**) Correspondence between fluorescence loss and gene deletion. The cell populations were collected from the 96-well plates, and the deletion of *mcherry-cat* gene was examined by PCR. We detected no bands of both *mcherry-cat* and *cat* for Samples 1–5. Sample 6 retained the *mcherry-cat* gene. The bottom plot shows that the absence of *mcherry-cat* gene corresponds to the loss of mCherry-CAT fluorescence. (**C**) MIC test. The OD_600_ values of Samples 1–6, YK0083 and YK0085 after a 23 hr incubation period at the indicated Cp concentrations are shown (Start OD_600_, 0.001). The entries with colored backgrounds indicate OD_600_>0.04.

**Figure 3—figure supplement 4.**
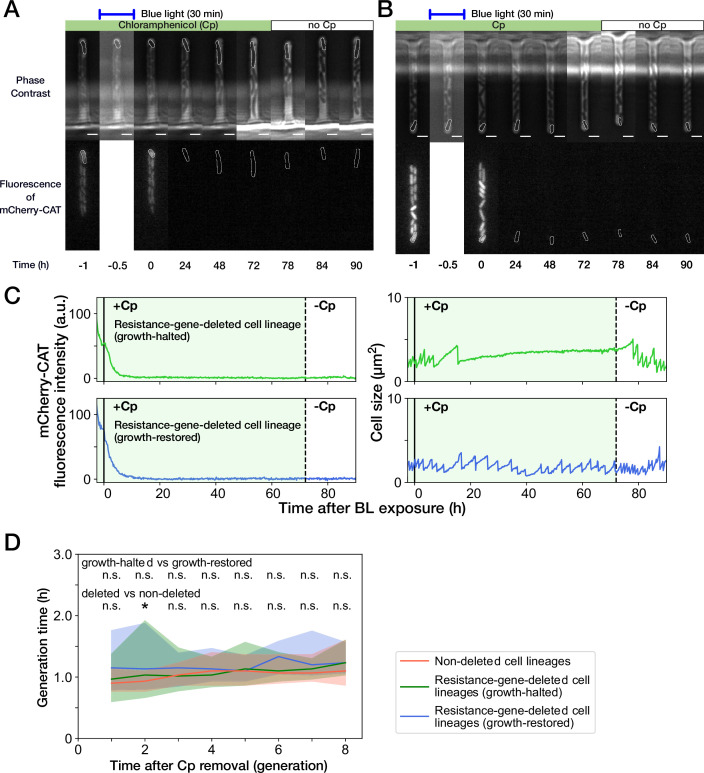
Growth recovery of resistance-gene-deleted cell lineages after Cp removal. (**A,B**) Representative time-lapse images of cell lineages that lost *mcherry-cat* resistance gene. (**A**) For growth-halted cell lineages; (**B**) for growth-restored cell lineages. The upper and lower sequences show phase contrast and mCherry fluorescence images, respectively. Blue light was illuminated from t = -0.5 hr to 0 hr. Cp was removed at t = 72 hr. The cells at the closed end of the growth channel were monitored during the experiment, which are outlined in white on the images. Scale bars, 2.5 µm. (**C**) Transitions of mCherry-CAT fluorescence intensities and cell size in single-cell lineages. The upper plots show the transitions in a growth-halted resistance-gene-deleted cell lineage; the lower plots show those of a growth-restored resistance-gene-deleted cell lineage. The solid black lines represent the end time of blue light illumination. The dashed black lines represent the time of Cp removal. (**D**) Transition of generation time after Cp removal. The lines and shaded areas represent the medians and the 25–75% ranges, respectively. The transitions are shown in generations after first cell divisions. Red, blue, and green represent non-deleted cell lineages, growth-restored resistance-gene-deleted cell lineages, and growth-halted resistance-gene-deleted cell lineages, respectively. The p-values of Mann-Whitney test were 0.11, 0.31, 0.45, 0.089, 0.50, 0.012, 0.038, and 0.27 for growth-halted vs growth-restored cell lineages for the generations from 1st to 8th, respectively. Also, the p-values were 0.057, 0.0070, 0.23, 0.48, 0.17, 0.032, 0.14, and 0.063 for resistance-gene-deleted vs non-deleted cell lineages. The p-values below 0.01 were assumed significant and indicated by asterisk.

**Figure 3—figure supplement 5.**
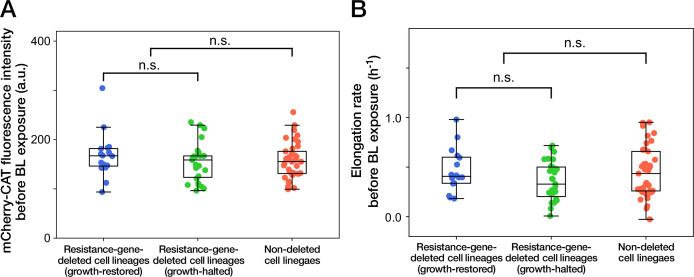
Cellular phenotypes before blue-light illumination do not correlate with the cellular fates. (**A**) mCherry-CAT fluorescence before blue-light illumination. Each point represents the fluorescence intensity of single-cell lineage averaged over the 1 hr period immediately before blue-light illumination. Blue points represent the growth-restored resistance-gene-deleted cell lineages. Green points represent the growth-halted resistance-gene-deleted cell lineages. Red points represent the non-deleted cell lineages. p = 0.30 for growth-restored resistance-gene-deleted cell lineages vs growth-halted resistance-gene-deleted cell lineages; p = 0.85 for resistance-gene-deleted cell lineages vs non-deleted cell lineages, Welch’s t-test. (**B**) Elongation rates before blue-light illumination. Each point represents the elongation rate of single-cell lineage averaged over the 1 h period immediately before blue-light illumination. The color correspondence remains the same as A. p = 0.12 for growth-restored resistance-gene-deleted cell lineages vs growth-halted resistance-gene-deleted cell lineages; p = 0.35 for resistance-gene-deleted cell lineages vs non-deleted cell lineages, Welch’s t-test.

**Video 2.** Growth restoration under Cp exposure after resistance gene deletion. YK0083 cells were cultured in the mother machine flowing the M9 minimal medium containing 15 µg/mL of Cp. Blue light was illuminated from t = –30 min to 0 min (marked with ‘ + BL’). The merged images of phase contrast (grayscale) and mCherry-CAT fluorescence (red) channels are shown. The illumination caused the loss of fluorescence signals, that is, the deletion of the *mcherry-cat* gene, in this cell lineage. Nevertheless, the cell gradually restored growth and division under continuous Cp exposure. Scale bar, 5 µm.

We first suspected that the growth recovery under Cp exposure was attributed to the presence of the *cat* gene untagged from the *mcherry* gene or by additional unintended resistance-enhancing mutations. To test this hypothesis, we illuminated batch cultures of YK0083 cells by blue light for 30 min under exposure to 15 µg/mL of Cp and plated them on agar plates. Colony PCR confirmed that all the cells that lost mCherry fluorescence were also *cat*-negative even when the deletion was introduced under Cp exposure (Figure 3—figure supplement 2A). This result strongly suggests that the growth-restored cells do not retain the *cat*-resistance gene as designed. The fraction of resistance-gene-deleted cells after 30-min blue-light illumination monotonically decreased in batch culture containing 15 µg/mL of Cp (Figure 3—figure supplement 2B), which is consistent with the observation that growth-restored resistance-gene-deleted cells grew more slowly than non-deleted cells.

In addition, we sampled the culture media flowing out from the microfluidic device and obtained cell populations derived from a single or few ancestral cells by limiting dilution (Figure 3—figure supplement 3A). PCR analysis confirmed the lack of *cat* gene in the non-fluorescent cell populations (Figure 3—figure supplement 3B). Furthermore, whole-genome sequencing of the cell populations obtained by limiting dilution showed no additional mutations in four out of five non-fluorescent cell populations tested (Table 1). One point mutation was present in one cellular population, but this mutation did not affect the MIC (Figure 3—figure supplement 3C). To calculate the probability that all of these five non-fluorescent cell populations had originated from resistance-gene-deleted growth-halted cells, we analyzed the regrowing dynamics of both growth-restored and growth-halted cell lineages after removing Cp in the microfluidic device (Figure 3—figure supplement 4A-C and Video 3). We found that 91.3% (73/80) of growth-restored cell lineages recovered fast growth after the exposure to 15 µg/mL of Cp for 72 hr. On the other hand, the proportion of cells that recovered growth was lower for the growth-halted cell lineages; only 69.3% (140/202) of growth-halted cell lineages could resume growth. The growth after first cell divisions were as fast as that for the non-deleted cells and was indistinguishable between the growth-restored and growth-halted cell lineages (Figure 3—figure supplement 4D). Taking the fraction of growth-restored cells among the resistance-gene-deleted cells and the proportions of growth-recovered cells after Cp removal into account, we found that the probability that all the five cell populations were derived from growth-halted cell lineages was 5.5% (see Materials and methods). Therefore, the probability that unintended genetic changes were responsible for the growth restoration is small.

**Table 1.** Mutations detected by whole-genome sequencing. We obtained isolated cellular populations derived from single or few cells by limiting dilution of the culture media flowing out from the mother machine. We detected only one point mutation in Sample 3.

Sample no.	Mutation	Position	Base	Annotation
Sample 1	No mutation			
Sample 2	No mutation			
Sample 3	Mutation in leuC site	80,471	G - > T	A132S
Sample 4	No mutation			
Sample 5	No mutation			

**Video 3.** Growth recovery of resistance-gene-deleted cells after Cp removal. Left, growth-halted resistance-gene-deleted cell; Right, Growth-restored resistance-gene-deleted cells. Cp was removed at t = 0 min (72 hr after blue light illumination). The merged images of phase contrast (grayscale) and mCherry-CAT fluorescence (red) channels are shown. Scale bar, 5 µm.

It is also unlikely that the growth restoration was caused by the residual mCherry-CAT proteins because 30 consecutive cell divisions dilute cytoplasmic proteins $2^{30}\approx 10^{9}$ folds if additional production is prevented; note that even the total number of protein molecules in a bacterial cell are in the order of 10^6^-10^7^ (Milo, 2013) We also detected no significant differences in the mCherry-CAT fluorescence intensity before blue-light illumination between the growth-halted and growth-restored cell lineages (Figure 3—figure supplement 5A). Furthermore, no significant differences were observed even between the resistance-gene-deleted and non-deleted cell lineages (Figure 3—figure supplement 5A). We also examined the influence of elongation rate before blue-light illumination on the gene deletion and the fates after gene deletion, finding no correlations (Figure 3—figure supplement 5B). Therefore, neither the amount of mCherry-CAT proteins at the time of gene deletion nor pre-deletion elongation rate affected the likelihood of gene deletion and the determination of growth-halt and growth-restoration fates under these experimental conditions.

We also found that deleting the *mcherry-cat* gene flowing a medium containing a twofold concentration of Cp (i.e., 30 µg/mL) eliminated growth-restored cell lineages: 33.1% (361/1092) of the cells illuminated by blue light lost the resistance gene, and none of them restored growth. This result suggests that deleting the resistance gene at the concentrations of Cp sufficiently higher than the MIC can prevent physiological adaptation.

### Resistance gene deletion long before Cp exposure prevents growth restoration

**Figure 4.**
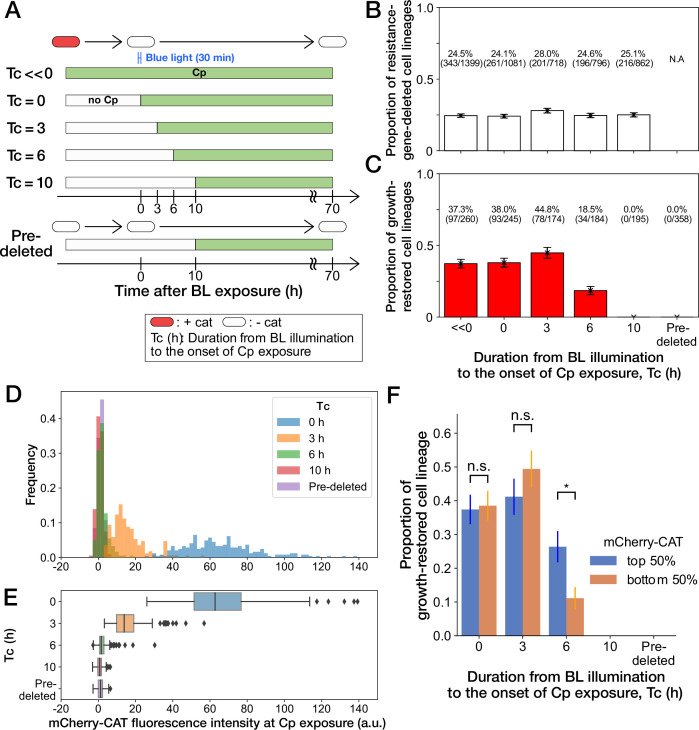
History-dependent maintenance of Cp resistance. (**A**) The schematic diagram of experiments. The duration from the end of blue-light illumination to the onset of Cp exposure ($T_{c}$) was varied from 0 to 10 h. $T_{c}\ll 0$ represents the continuous Cp-exposure condition. “Pre-deleted” denotes the results of the experiments performed with the *mcherry-cat*-deleted YK0085 strain. The non-resistant YK0085 cells were not exposed to blue light. (**B**) Fractions of *mcherry-cat*-deleted cell lineages across different $T_{c}$ conditions. The numbers of resistance-gene-deleted cell lineages among the total numbers of cell lineages observed during the measurements are shown above the bars. Error bars represent standard errors. (**C**) Fractions of growth-restored cell lineages among resistance-gene-deleted cell lineages. The numbers of growth-restored cell lineages among all the resistance-gene-deleted cell lineages are shown above the bars. Error bars represent standard errors. The numbers of resistance-gene-deleted cell lineages are different from those in B because some cell lineages were flushed away from the growth channels at the later time points of the measurements, and their fates could not be determined. (**D, E**) The distributions of the mCherry-CAT fluorescence intensities at the onset of Cp exposure across the different $T_{c}$ conditions represented by histograms (**D**) and box plots (**E**). (**F**) Fractions of growth-restored cell lineages and their dependence on the mCherry-CAT fluorescence at the onset of Cp exposure. Blue and orange bars indicate the fractions of growth-restored cells among cell lineages whose mCherry-CAT fluorescence intensities were higher and lower than the median, respectively. Error bars represent standard errors. The growth-restored cell lineages were not detected under the $T_{c}$ = 10 h and “Pre-deleted” conditions. Fractional differences between the top 50% and the bottom 50% of cell lineages were statistically significant only for the $T_{c}$ = 6 h condition (p = 0.86 for $T_{c}$ = 0 hr; p = 0.28 for $T_{c}$ = 3 hr; and p = 8.6 × 10^–3^ for $T_{c}$ = 6 hr, two proportional z-test).

The high frequency of growth restoration observed against the resistance gene deletion was unexpected since the MIC of the susceptible strain was below 15 µg/mL (Figure 2 and Figure 3—figure supplement 3C). To understand this discrepancy, we cultured the susceptible YK0085 cells in the mother machine first without Cp and then exposed them to 15 µg/mL of Cp directly in the device. In contrast to the previous observation, all cells stopped growth and division entirely, with no cells restoring growth (“Pre-deleted” in Figure 4A–C and Video 4). This result excludes the hypothesis that the unique cultivation environments in the microfluidics device are the cause of growth restoration. Instead, this observation implies that the timing of gene deletion is crucial for the cells to withstand the Cp exposure without the resistance gene and restore growth.

**Video 4.** No growth restoration of YK0085 strain under Cp exposure. YK0085 cells were cultured in the mother machine flowing the M9 minimal medium and exposed to 15 µg/mL of Cp at t = 0 min (marked with ‘ + Cp’). The Cp exposure caused growth arrest, and, unlike the YK0083 cells, the cell did not restore growth. We found no growth-restored cell lineages in this experimental condition. Scale bar, 5 µm.

To further investigate the importance of the timing of gene deletion, we performed microfluidic single-cell measurements with the resistant YK0083 strain, varying the duration from blue-light illumination to the onset of Cp exposure ($T_{c}$) between 0 h to 10 h (Figure 4A). The proportions of cells that lost the *mcherry-cat* gene in response to blue-light illumination almost remained unchanged among all conditions, ranging from 24% to 28% (Figure 4B). However, the proportions of growth-restored cell lineages among resistance-gene-deleted cells strongly depended on $T_{c}$ (Figure 4C). When Cp exposure was initiated immediately after the blue-light illumination ($T_{c}=$ 0 hr) or after 3 h ($T_{c}=$ 3 hr), the proportions of growth-restored cells were nearly equivalent to those observed under continuous Cp exposure conditions (Figure 4C). In contrast, the proportions of growth-restored cells were reduced to 18.5% (34/184) when $T_{c}=6$ hr, and we did not detect any growth restoration when $T_{c}$ = 10 hr (0/195; Figure 4C). The almost equivalent frequencies of growth-restored cell lineages under the $T_{c}$ = 0 hr and $T_{c}$ = 3 hr conditions and those observed under continuous exposure exclude the possibility that growth restoration requires prior exposure to Cp before resistance gene deletion.

We next conjectured that a low amount of mCherry-CAT proteins is required at the onset of Cp exposure to withstand and restore growth without the resistance gene. In fact, *mcherry-cat* gene deletion before Cp exposure led to the dilution of mCherry-CAT proteins by growth by the time of Cp exposure (Figure 4D and E).

To examine whether the mCherry-CAT concentration in individual cells affects growth restoration, we divided the resistance-gene-deleted cell lineages into two groups under each condition (top 50% and bottom 50%) based on their mCherry-CAT fluorescence intensities at the onset of Cp exposure. While we found no significant differences in the $T_{c}$ = 0 and 3 hr conditions, the top 50% group produced 2.4-fold more growth-restored cells than the bottom 50% group in the $T_{c}$ = 6 hr condition (Figure 4F). In the $T_{c}$ = 10 hr condition, we could barely detect the fluorescence signals from any cells (Figure 4D and E), and no cells showed growth restoration as mentioned above (Figure 4F). These results suggest that a low level of residual mCherry-CAT proteins is required at the onset of Cp exposure, but high amounts do not necessarily ensure the growth restoration in all resistance-gene-deleted cells. Moreover, 40% could be considered as the maximum frequencies at which cells can restore growth without the resistance gene.

### Growth restoration accompanies the recovery of stoichiometric balance of ribosomal subunits

The results above demonstrated that the residual mCherry-CAT proteins are important for initiating the transition to growth restoration without the resistance gene. However, the residual proteins cannot directly support the growth in later generations under Cp exposure as their levels are eventually diluted with growth. Indeed, the amounts of mCherry-CAT proteins during growth restoration were below the detection limit (Figure 3F and H). Therefore, we speculated that the other physiological changes were responsible for growth restoration.

**Figure 5.**
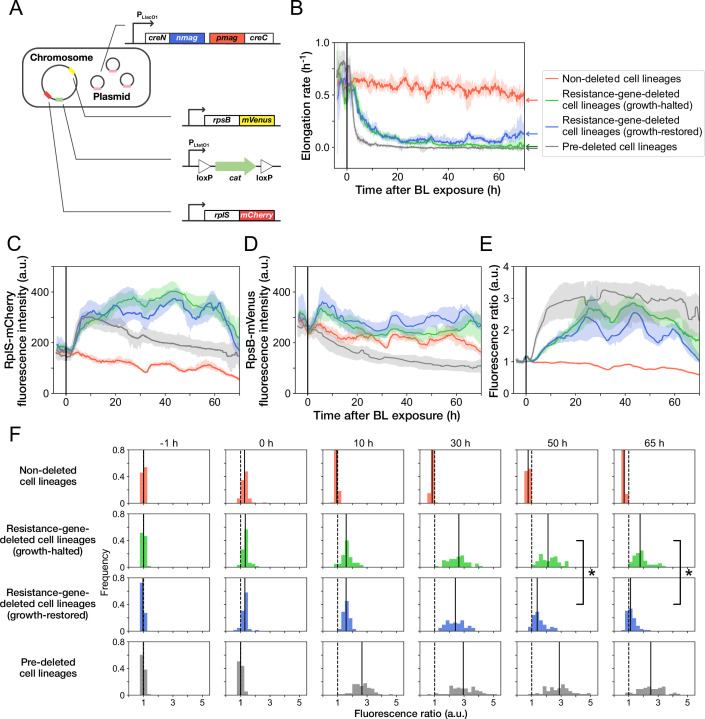
Disruption and restoration of ribosomal proteins’ stoichiometry. (**A**) Schematic diagram of the ribosome reporter strain, YK0136. Fluorescently tagged ribosomal protein genes (*rplS* and *rpsB*) are expressed from the native loci on the chromosomes. Additionally, the photo-removable *cat* gene was integrated into the *intC* locus on the chromosome. The PA-Cre fragments were expressed via low-copy plasmids. (**B**) Transitions of elongation rates. Lines represent the medians of elongation rates at each time point, and shaded areas show the 95% error ranges of the medians estimated by resampling the cell lineages 1000 times. Red represents non-deleted cell lineages. Green shows growth-halted resistance-gene-deleted cell lineages. Blue represents growth-restored resistance-gene-deleted cell lineages. Gray represents pre-deleted cell lineages. Color correspondence remains the same in the following figures. The end of blue-light illumination was set to 0 hr on the horizontal axis. (**C–E**) Transitions of RplS-mCherry fluorescence intensities (**C**), RpsB-mVenus fluorescence intensities (**D**), and the fluorescence ratio of RplS-mCherry/RpsB-mVenus (**E**). The fluorescence intensities of RplS-mCherry and RpsB-mVenus were normalized by the intensity observed before blue light illumination to calculate the ratio in E. (**F**) Transitions of the RplS-mCherry/RpsB-mVenus fluorescence ratio distributions. The vertical dashed lines represent the position of ratio = 1; the solid lines represent the medians of the distributions. Significant differences in fluorescence ratio were detected between the growth-restored and growth-halted cell lineages at 50 hr and 65 hr (p = 5.6 × 10 ^–10^ at 50 hr, p = 1.9 × 10 ^–13^ at 65 hr, Mann-Whitney U test).

Because Cp targets the 50 S ribosomal subunit (Pongs, 1979), it is plausible that ribosomal states were modulated in the duration beginning from resistance gene deletion to growth restoration. Hence, we constructed an *E. coli* strain YK0136 that expressed fluorescently-tagged dual ribosomal reporters, RplS-mCherry and RpsB-mVenus (Figure 5A; Nikolay et al., 2014). RplS and RpsB are ribosomal proteins in the 50 S and 30 S subunits, respectively. Their fluorescent reporters were utilized to probe the ribosomal subunit balance in living cells (Nikolay et al., 2014). YK0136 also harbors the *cat* resistance gene (not tagged with *mcherry*) between the upstream and downstream *loxP* sequences on the chromosome, and the PA-Cre recombination system was expressed via the plasmid (Figure 5A). We confirmed that YK0136 showed MIC values almost equivalent to those of the YK0083 strain (Figure 2 and Figure 5—figure supplement 1). Furthermore, 30-min blue-light illuminations provoked *cat*-gene deletion in 28.6% (452/1587) of YK0136 cells, which was comparable to the frequency of *mcherry-cat*-gene deletion in YK0083 (Figure 4B).

**Figure 5—figure supplement 1.**
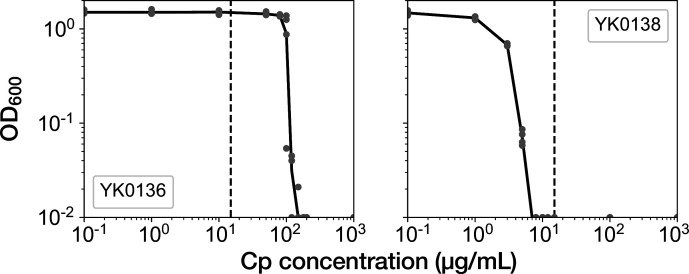
MIC tests of the ribosome reporter strains. Gray points indicate the OD_600_ of the cell cultures after a 23 hr incubation period in the media containing the corresponding concentrations of Cp. The left shows the result of YK0136. The right shows the result of YK0138. The minimum concentration where OD_600_ became lower than 0.01 was adopted as the MIC for each strain. 15 µg/mL of Cp was used in the experiments (dashed line). The measurements were repeated at least thrice.

**Figure 5—figure supplement 2.**
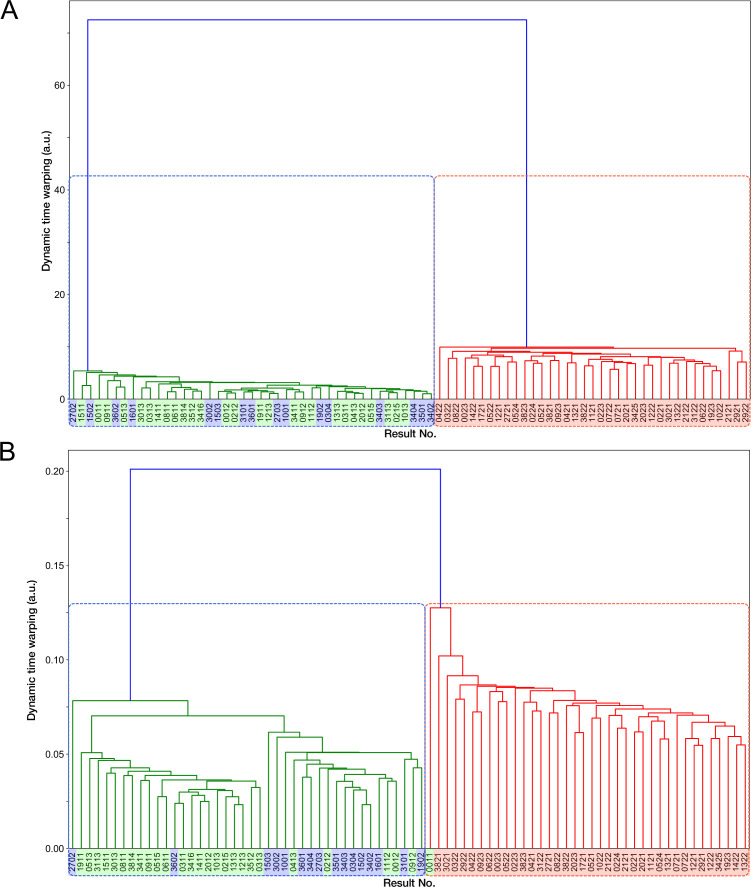
Time-course transitions of elongation rates are sufficient for classifying resistance-gene-deleted and non-deleted cell lineages. (**A**) Hierarchical clustering of single-cell lineages based on the transitions of mCherry-CAT fluorescence intensities. The mCherry-CAT fluorescence transitions in single-cell lineages over the entire periods of measurement were used for clustering. The distances between cell lineages were calculated by dynamic time warping. The numbers shown on the horizontal axis represent cell lineage IDs. The background colors behind the cell lineage IDs represent the fates of cell lineages assigned manually by the experimenters: Blue corresponds to growth-restored resistance-gene-deleted cell lineages; Green represents growth-halted resistance-gene-deleted cell lineages; Red represents non-deleted cell lineages. (**B**) Hierarchical clustering of single-cell lineages based on the transitions of elongation rates. The cell lineage clusters indicated by the blue and red dotted lines constitute two main groups, corresponding to resistance-gene-deleted and non-deleted cell lineages, respectively. Only one resistance-gene-deleted cell lineage (0011) was assigned wrongly as a non-deleted cell lineage. Therefore, the transitions of elongation rates are sufficient for classifying the resistance-gene-deleted and non-deleted cell lineages at high precision.

**Figure 5—figure supplement 3.**
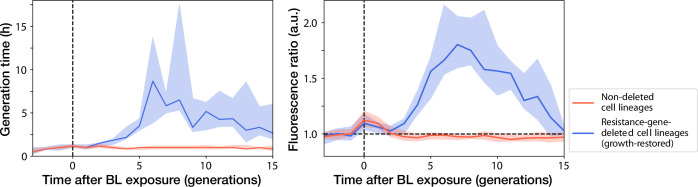
Transitions of generation time and RplS-mCherry/RpsB-mVenus fluorescence ratio observed in ribosome reporter strain shown in generation. Left: Generation time transitions; Right: RplS-mCherry/RpsB-mVenus fluorescence ratio transitions. The lines and shaded areas represent the medians and the 25%–75% data ranges, respectively. Blue corresponds to growth-restored resistance-gene-deleted cell lineages. Red corresponds to non-deleted cell lineages.

**Figure 5—figure supplement 4.**
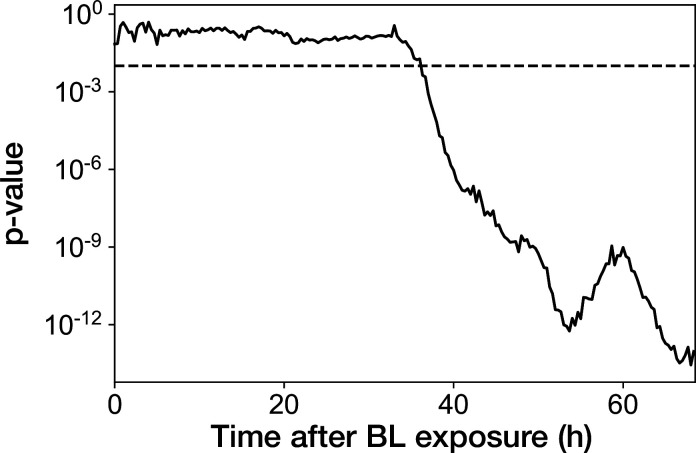
Transition of p-value from the Mann-Whitney U test applied for the RplS-mCherry/RpsB-mVenus fluorescence ratio. Mann-Whitney U test was performed for the fluorescence ratio between growth-restored resistance-gene-deleted cell lineages and growth-halted resistance-gene-deleted cell lineages at each time point after blue-light illumination. Dashed horizontal line represents the p-value of 0.01.

**Figure 5—figure supplement 5.**
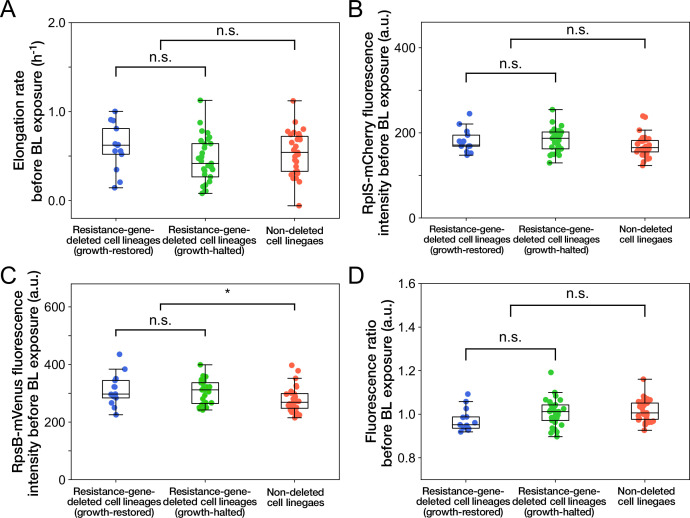
Relationships between cellular phenotypes before blue-light illumination and cellular fates. (**A**) Relationship between elongation rates before blue-light illumination and cellular fates. Blue represents growth-restored resistance-gene-deleted cell lineages. Green represents growth-halted resistance-gene-deleted cell lineages. Red represents non-deleted cell lineages (Color correspondence remains the same in the following figures). Each point represents the elongation rate of single-cell lineage averaged over the 1 hr period immediately before blue-light illumination. p = 0.10 for growth-restored vs growth-halted cell lineages; p = 0.54 for resistance-gene-deleted vs non-deleted cell lineages, Welch’s t-test. (**B**) Relationship between RplS-mCherry fluorescence intensities averaged over the 1 hr period before blue-light illumination and cellular fates. p = 0.87 for growth-restored vs growth-halted cell lineages; p = 0.038 for resistance-gene-deleted vs non-deleted cell lineages, Welch’s t-test. (**C**) Relationship between RpsB-mVenus fluorescence intensities averaged over the 1 hr period before blue-light illumination and cellular fates. p = 0.60 for growth-restored vs growth-halted cell lineages; p = 0.010 for resistance-gene-deleted vs non-deleted cell lineages, Welch’s t-test. Asterisk denotes the statistical difference judged at the significance level of 0.01. (**D**) Relationship between RplS-mCherry/RpsB-mVenus fluorescence ratio averaged over the 1 hr period before blue-light illumination and cellular fates. p = 0.098 for growth-restored vs growth-halted cell lineages; p = 0.15 for resistance-gene-deleted vs non-deleted cell lineages, Welch’s t-test.

We conducted time-lapse measurements of YK0136 cells in the mother machine and induced the deletion of the resistance gene via blue-light illumination under continuous exposure to 15 µg/mL of Cp (Video 5). Since previous experiments showed that the resistance gene deletion decelerates cellular growth significantly, the deletion of the *cat* gene in each cell lineage was evaluated based on the distinctive declines of cellular elongation rates after the blue-light illumination. We validated this criterion by hierarchical time-series clustering applied to the growth of YK0083 cells and mCherry-CAT fluorescence transitions (Figure 5—figure supplement 2 and Materials and methods).

**Video 5.** Recovery of the balance of ribosomal proteins alongside growth restoration. YK0136 cells were cultured in the mother machine flowing the M9 minimal medium containing 15 µg/mL of Cp. Blue light was illuminated from t = –30 min to 0 min (marked with ‘ + BL’). The merged images of phase contrast (grayscale), RplS-mCherry fluorescence (red), and RpsB-mVenus fluorescence (green) channels are shown. The growth of the cell markedly slowed down after blue-light illumination, indicating the loss of the *cat* gene. The cell became red in response to blue-light illumination, indicating that the relative expression level of RplS-mCherry became higher than that of RpsB-mVenus. However, the balance was restored alongside growth restoration. Scale bar, 5 µm.

As observed previously, 45.9% (158/344) of the *cat*-deleted YK0136 cells gradually restored their growth after initial growth suppression (Figure 5B, Figure 5—figure supplement 3, and Video 5). Furthermore, such growth-restored cell lineages were not observed (0/1138) when YK0138 cells were exposed to 15 µg/mL of Cp; the YK0138 strain was constructed from YK0136 cells by deleting the *cat* gene beforehand in batch culture via blue-light illumination (Figure 5B and Video 6).

**Video 6.** No recovery of the balance of ribosomal proteins in the YK0138 strain. YK0138 cells were cultured in the mother machine flowing the M9 minimal medium and exposed to 15 µg/mL of Cp at t = 0 min (marked with ‘ + Cp’). The Cp exposure caused growth arrest and disruption of ribosomal proteins’ expression balance. No restoration of growth and ribosomal balance was observed. Scale bar, 5 µm.

The transitions of fluorescence intensities reveal that *cat*-gene deletion under Cp exposure increased the expression levels of both RplS-mCherry and RpsB-mVenus (Figure 5C and D). The relative changes triggered by deletion were more significant for RplS-mCherry than those for RpsB-mVenus (Figure 5C and D). Consequently, the ribosomal subunit balance probed by the RplS-mCherry/RpsB-mVenus ratio was disrupted, and the ratio increased approximately 3-fold (Figure 5E and F). The initial disruption kinetics was similar between growth-restored and growth-halted resistance-gene-deleted cell lineages (Figure 5E). However, the ratio of growth-restored cell lineages gradually returned to the original level, whereas the ratio of growth-halted cell lineages remained disrupted (Figure 5E and F). The difference in the ratio between growth-restored and growth-halted cell lineages became evident approximately 37 hr after resistance gene deletion (Figure 5—figure supplement 4). These results suggest that the regain of the ribosomal subunit balance under Cp exposure is correlated with growth restoration.

Analysis of the correlations between the pre-illumination phenotypic traits and post-illumination cellular fates reveals that elongation rates, fluorescence intensities of RplS-mCherry and RpsB-mVenus, and the fluorescence ratio observed before blue-light illumination did not strongly affect the likelihood of gene deletion and the determination of growth-halting and growth-restoration fates (Figure 5—figure supplement 5).

When non-resistant YK0138 cells were exposed to 15 µg/mL of Cp, the expression levels of RplS-mCherry increased initially following kinetics similar to those of the YK0136 cells observed after *cat*-gene deletion (Figure 5C). In contrast, the expression levels of RpsB-mVenus decreased in response to Cp exposure (Figure 5D), as previously reported for a wildtype *E. coli* strain exposed to Cp (Siibak et al., 2011). The initial decrease of RpsB-mVenus expression led to a more rapid disruption of the RplS-mCherry/RpsB-mVenus ratio in the susceptible YK0138 cells (Figure 5E and F). Consistently, the decline in the elongation rates of the YK0138 cells was faster than those of the YK0136 cells (Figure 5B).

**Figure 6.**
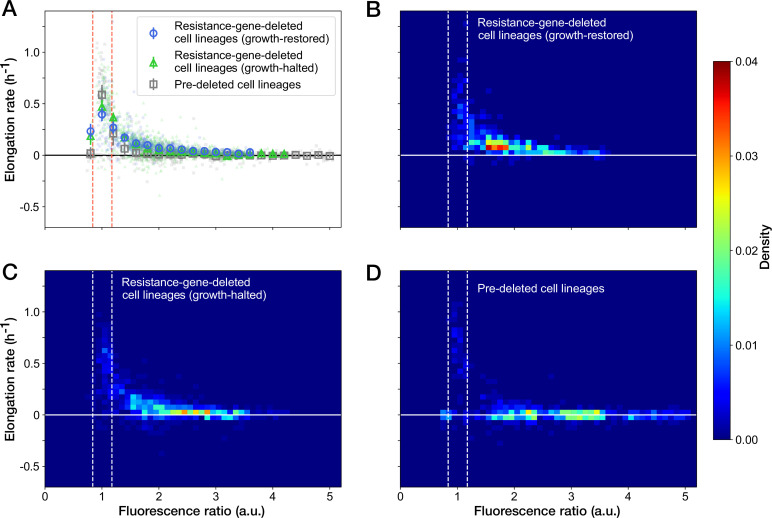
Relationship between the RplS-mCherry/RpsB-mVenus fluorescence ratio and elongation rate under Cp exposure. (**A**) Scatter plot of the RplS-mCherry/RpsB-mVenus fluorescence ratio and elongation rate. Small points represent the relations between the fluorescence ratio and elongation rates averaged over the two-hour periods at the different time points on the single-cell lineages. The larger open point represents the mean elongation rate in each bin of fluorescence ratio (bin width, 0.2 a.u.). Error bars represent the 1.96× standard error ranges. Blue represents growth-restored resistance-gene-deleted cell lineages. Green represents growth-halted resistance-gene-deleted cell lineages. Gray represents pre-deleted cell lineages. Dashed lines indicate the mean ± 1.96× standard error range of fluorescence ratio of non-deleted cell lineages before blue-light illumination. (**B–D**) Density plots of the points for the relationships between fluorescence ratio and elongation rate. (**B**) Growth-restored resistance-gene-deleted cell lineages. (**C**) Growth-halted resistance-gene-deleted cell lineages. (**D**) Pre-deleted cell lineages. Color represents the density of the points for each type of cell lineages shown in (**A**). Bin size was 0.05 hr^-1^ for elongation rate and 0.1 a.u. for fluorescence ratio.

We further examined the relationship between the RplS-mCherry/RpsB-mVenus ratio and elongation rates and observed a negative correlation (Spearman ρ = –0.58, 95% confidence interval [-0.62,–0.53]; Figure 6A). The ratio was maintained within a narrow range (0.84–1.18, 95% interval) before blue-light illumination (Figures 5F and 6A). The disruption of ribosomal subunit balance exceeding this range was linked to growth suppression (Figure 6A). The relationships between the ratio and elongation rates were similar among growth-restored resistance-gene-deleted cell lineages, growth-halted resistance-gene-deleted cell lineages, and pre-deleted cell lineages (Figure 6A). However, the distribution of the points was shifted toward the original fluorescence ratio for the growth-restored resistance-gene-deleted cell lineages (Figure 6B–D). These results suggest that the ribosomal subunit balance is correlated with growth under Cp exposure and that restoration of the subunit balance might help cells to recover growth in the absence of the resistance gene.

## Discussion

Uncovering the genotype-phenotype correspondences in various biological contexts is the groundwork for genetics. However, the correspondences are usually investigated based on terminal phenotypes, which are often manifested after significant lags following genotypic changes. Consequently, our understanding of the dependence of terminal phenotypes on historical conditions and the heterogeneity of phenotypic consequences among individual cells remains limited. In this study, we demonstrated that *E. coli* cells could adapt even to a lethal genetic modification, that is the deletion of *cat* resistance gene under exposure to chloramphenicol (Figure 3). Importantly, such adaptation was not observed when an identical genetic modification was introduced long before Cp exposure (Figure 4). Therefore, whether cells could gain physiological resistance in the absence of a resistance gene depends on the timing of resistance gene deletion and environmental histories that the cells experienced.

The analyses revealed that the *cat* gene deletion under Cp exposure disrupted the stoichiometric balance of ribosomal proteins (RplS and RspB) and that the balance was gradually recovered alongside growth restoration (Figure 5). The elongation rate and the stoichiometric balance of ribosomal proteins were strongly correlated (Figure 6). Furthermore, the balance was tightly regulated within a narrow range in the fast-growing non-deleted cells (Figure 5F). Therefore, the recovery of ribosomal stoichiometric balance might contribute to restoring growth against the deletion of the resistance gene.

It still remains elusive how individual cells recovered the ribosomal stoichiometric balance under continuous Cp exposure. A plausible scenario might be that the multi-layered and interlinked feedback regulations on ribosomal components entailed stoichiometry readjustments (Yamagishi and Nomura, 1988; Nomura et al., 1984; Lindahl and Zengel, 1986; Keener and Nomura, 1996; Kaczanowska and Rydén-Aulin, 2007). Many ribosomal proteins bind to their own mRNAs, in addition to the target ribosomal RNAs (Nomura et al., 1984; Lindahl and Zengel, 1986; Keener and Nomura, 1996; Kaczanowska and Rydén-Aulin, 2007). These regulatory fractions of ribosomal proteins bind to their mRNAs and inhibit their own translation and that of other genes in the same operons. The competition among rRNA and mRNA for these regulatory ribosomal protein components is considered to regulate the stoichiometry of ribosomal components (Nomura et al., 1984; Lindahl and Zengel, 1986; Keener and Nomura, 1996; Kaczanowska and Rydén-Aulin, 2007). Therefore, it is plausible that these innate homeostatic mechanisms of ribosomes might have been exploited by cells to restore growth. It is also important to note that the operons for ribosomal proteins comprise genes encoding RNA polymerase subunits, translation initiation/elongation factors, DNA primase and protein transmembrane transporters (Lindahl and Zengel, 1986; Keener and Nomura, 1996). Hence, the feedback regulations in ribosomes could mediate changes in the global expression profiles, which might have helped the cells find growth-permissive states in the presence of Cp.

The necessity for a small amount of residual resistant proteins at the onset of Cp exposure (Figure 4) might suggest that experiencing such barely-tolerable intracellular states is crucial for gaining physiological resistance. The importance of near-critical states for survival might be related to the phenomenon that pre-treatment with a sublethal concentration of antibiotics increases the fraction of persister cells in response to the subsequent treatments with lethal antibiotic concentrations (Dörr et al., 2009; Johnson and Levin, 2013). We also remark that the physiological adaptation to the antibiotic stress caused by resistance gene deletion might be related to *adaptive resistance*. Adaptive resistance is a phenomenon in which bacterial populations progressively enhance their resistance levels (MICs) when subject to gradual increases of antibiotics (George and Levy, 1983; Adam et al., 2008; Sánchez-Romero and Casadesús, 2014; Motta et al., 2015). Adaptive resistance is achieved physiologically rather than genetically since the enhanced resistance is lost when the antibiotic is removed. Therefore, it is plausible that growth-restoration against resistance gene deletion might be realized by some mechanisms common to adaptive resistance. The fact that no cells recovered growth when the resistance gene was deleted at a high Cp concentration (30 µg/mL) also suggests a link to adaptive resistance; the speed of antibiotic stress intensification must be sufficiently slow for the cells to gain resistance physiologically. However, we also remark that it is nontrivial whether bacterial cells respond to the intensification of antibiotic stress caused by internal resistance-gene deletion similarly to the stress caused by gradual increases of external antibiotic concentrations. Although our results might support the hypothesis that physiological adaptation to genetic perturbations that sensitize cells to some antibiotics is feasible when bacterial cells exhibit adaptive resistance to those drugs, more careful examinations under diverse experimental conditions should be needed to understand the identity of these internally and externally intensified antibiotic stress. Additionally, we note that adaptive resistance has been investigated at the population level, not at the single-cell level, in the previous studies (George and Levy, 1983; Adam et al., 2008; Sánchez-Romero and Casadesús, 2014). Consequently, it remains elusive whether only a small fraction of cell lineages in the populations acquire resistance and dominate the populations by selection, or most cells can progressively enhance resistance in adaptive resistance. In this study, using the mother machine microfluidic device, we were able to quantify the fractions of cell lineages that restore growth against resistance-gene deletion and the changes of the fractions depending on the historical conditions. Such single-cell-level quantitative information would be valuable for unraveling the windows of phenotypic space and the ranges of conditions permitting physiological adaptation to antibiotics.

Though experimental evidence of history-dependent physiological adaptation to lethal genetic perturbations is still limited, the experimental strategy combining optogenetic recombination and single-cell lineage tracking is applicable for the modification of other genes or other cell types, including mammalian cells. Such approaches might offer new insights into the emergence of drug-resistant bacterial and cancer cells. We remark that growth-restoration of resistance-gene-deleted cells must have been hardly recognized in batch-culture experiments because the fractions of these slow-growing cells decrease in time by selection within cell populations (Figure 3—figure supplement 2B). Therefore, our study also confirms the advantage of microfluidic time-lapse microscopy for unraveling long-term adaptation phenomena that occur in slow-growing cell lineages. Gaining a more comprehensive picture of resistance evolution might help us design new drug treatment strategies to counter or control the emergence and spread of resistance.

## Materials and methods

**Key resources table:** 

Reagent type (species) or resource	Designation	Source or reference	Identifiers	Additional information
Strain,strain background (*Escherichia coli*)	F3	[31]		W3110 Δ*fliC*::FRT Δ*fimA*::FRT Δ*flu*::FRT
Strain,strain background (*Escherichia coli*)	YK0083	This study		F3 *intC*::P*_LtetO1_*-loxP-RBS4-*mcherry-cat-*loxP-FRT/pYK022
Strain,strain background (*Escherichia coli*)	MUS6	This study		F3 *rplS-mcherry*-FRT *rpsB-mvenus*-FRT
Strain,strain background (*Escherichia coli*)	YK0136	This study		MUS6 *intC*::P*_LtetO1_*-loxP-RBS4-*cat-*loxP-FRT/pYK022
Recombinant DNA reagent	*pmag-creC*	[12]		gifted from Sato lab.
Recombinant DNA reagent	*nmag-creN*	[12]		gifted from Sato lab.
Recombinant DNA reagent	pYK022	This study		plasmid expressing PA-Cre genes.
Sequence-based reagent	intC_check_F	This study	PCR primerfor intC check	GATCGATACTTG CTGTGGTTGATG
Sequence-based reagent	intC_check_R2	This study	PCR primerfor intC check	CCTCTTAGTTAAATG GATATAACGAGCCCC
Sequence-based reagent	YKp0077	This study	PCR primerfor cat check	CACCGTTGATA TATCCCAATGGC
Sequence-based reagent	YKp0115	This study	PCR primerfor cat check	CACTCATCG CAGTACTGTTG
Commercial assay or kit	Wizard SVGel and PCRClean-Up System	Promega	Cat.# A9281	
Commercial assay or kit	PureYieldPlasmid MiniprepSystem	Promega	Cat.# A1223	
Commercial assay or kit	WizardGenomic DNAPurification Kit	Promega	Cat.# A1120	
Chemical compound, drug	chloramphenicol	Wako	030-19452	
Chemical compound, drug	isopropyl-β-D-thiogalactopyranoside	Wako	094-05144	
Chemical compound, drug	Bovine Serum Albumin	Sigma-Aldrich	Cat.# A6003	
Chemical compound, drug	polydimethylsiloxane	Dow Corning	SYLGARD 184	
Software, algorithm	FastDTW	[32]		
Software, algorithm	HyperStackReg	[33]		

### Bacterial strains, plasmids construction, and culture conditions

*E. coli* strains, plasmids, and primers are listed in Table 2, Table 3 and Table 4, respectively. For strain constructions, cells were grown in Luria Bertani (LB) broth (Difco) at 37 ºC unless stated otherwise. Plasmids were constructed using DNA ligase (T4 DNA Ligase, Takara) or by ExoIII cloning (Li and Evans, 1997) and introduced into the *E. coli* strain JM109 for cloning and stocking. The *cat* or *mcherry-cat* gene was introduced into the *intC* locus of F3 (W3110*ΔfliC*::FRT*ΔfimA*::FRT*Δflu*::FRT) genome by λ-Red recombination (YK0080, YK0134) (Datsenko and Wanner, 2000). Fluorescently-tagged ribosome reporter genes were introduced into the *E. coli* strain BW25113 (MUS3, MUS13). These ribosome reporter genes were transferred into the F3 strain by P1 transduction (MUS5, MUS6). The colonies with intended genome integration were selected on LB plates containing kanamycin (Km, Wako). The Km resistant gene was removed by flp-FRT recombination using pCP20 plasmid (Datsenko and Wanner, 2000).

**Table 2.** Strain list used in this study.

mCherry-CAT deletion		
Name	Genotype	Sourse
F3	W3110 Δ*fliC*::FRT Δ*fimA*::FRT Δ*flu*::FRT	Hashimoto et al., 2016
YK0080	F3 *intC*::P_*LtetO1*_-loxP-RBS4-*mcherry-cat*-loxP-FRT	This study
YK0083	F3 *intC*::P_*LtetO1*_-loxP-RBS4-*mcherry-cat*-loxP-FRT/pYK022	This study
YK0085	F3 *intC*::P_*LtetO1*_-FRT/pYK022	This study
Ribosome reporter		
Name	Genotype	Sourse
MUS3	BW25113 *rplS-mcherry*-FRT-*kan*-FRT	This study
MUS13	BW25113 *rpsB-mvenus*-FRT-*kan*-FRT	This study
MUS5	F3 *rplS-mcherry*-FRT	This study
MUS6	F3 *rplS-mcherry*-FRT *rpsB-mvenus*-FRT	This study
YK0134	MUS6 *intC*::P_*LtetO1*_P-loxP-RBS4-*cat*-loxP-FRT	This study
YK0136	MUS6 *intC*::P_*LtetO1*_-loxP-RBS4-*cat*-loxP-FRT/pYK022	This study
YK0138	MUS6 *intC*::P_*LtetO1*_-loxP-FRT/pYK022	This study

**Table 3.** Plasmid list used in this study.

Name	Backbone	Gene	Sourse
pKD46			CGSC
pCP20			CGSC
	pcDNA3.1	pCMV-lox2272-loxP-inversed *mvenus*-lox2272-loxP	Sato lab
	pcDNA3.1	pCMV-rfp-*pmag-creC*	Sato lab
	pcDNA3.1	pCMV-rfp-*nmag-creN*	Sato lab
pTmCherryK3	pMW118	P_*LtetO1*_ -RBS3-*mcherry*-FLP-*kan*-FLP	Wakamoto lab
pLVK4	pMW118	P_*LlacO1*_ -RBS4-*mvenus*-FLP-*kan*-FLP	Wakamoto lab
pTVCK4	pMW118	P_*LtetO1*_ -RBS4-*mvenus-cat*-FLP-*kan*-FLP	Wakamoto lab
pKK1	pMW118	P_*LlacO1*_-rfp-*pmag-creC*-FLP-*kan*-FLP	This study
pKK2	pMW118	P_*LlacO1*_ -rfp-*nmag-creN*-FLP-*kan*-FLP	This study
pYK001	pMW118	P_*LlacO1*_-RBS3-*pmag-creC*-FLP-*kan*-FLP	This study
pYK002	pMW118	P_*LlacO1*_-RBS3-*nmag-creN*-FLP-*kan*-FLP	This study
pYK006	pMW118	P_*LtetO1*_-RBS3-lox2272-*mvenus*-loxP-FLP-*kan*-FLP	This study
pYK007	pMW118	P_*LtetO1*_-RBS3-lox2272-*mcherry*-loxP-FLP-*kan*-FLP	This study
pYK008	pMW118	P_*LtetO1*_-RBS3-lox2272-*mcherry*-loxP-FLP-*kan*-FLP	This study
pYK009	pMW118	P_*LtetO1*_-RBS3-lox2272-*mcherry*-loxP-FLP-*kan*-FLP	This study
pYK010	pMW118	P_*LtetO1*_-RBS4-lox2272-*mcherry*-loxP-FLP-*kan*-FLP	This study
pYK011	pMW118	P_*LtetO1*_-lox2272-RBS4-*mcherry*-loxP-FLP-*kan*-FLP	This study
pYK016	pMW118	P_*LlacO1*_-RBS3-*pmag-creC*-FLP-*kan*-FLP	This study
pYK017	pMW118	P_*LlacO1*_-RBS3-*creN-nmag*-FLP-*kan*-FLP	This study
pYK018	pMW118	P_*LlacO1*_-RBS3-*creN-nmag-pmag-creC*-FLP-*kan*-FLP	This study
pYK022	pMW118	P_*LlacO1*_-RBS3-*creN-nmag-pmag-creC*-FLP-*kan*-FLP	This study
pYK023	pMW118	P_*LtetO1*_-loxP-RBS4-*mcherry*-loxP-FLP-*kan*-FLP	This study
pYK028	pMW118	P_*LtetO1*_-loxP-RBS4-*mcherry*-loxP-FLP-*kan*-FLP	This study
pYK029	pMW118	P_*LtetO1*_-loxP-RBS4-*mcherry-cat*-loxP-FLP-*kan*-FLP	This study
pYK035	pMW118	P_*LtetO1*_-loxP-RBS4-*cat*-loxP-FLP-*kan*-FLP	This study
pMU1	pMW118	P_*LlacO1*_-RBS4-*mcherry*-FLP-*kan*-FLP	This study
pMU2	pMW118	P_*LlacO1*_-RBS4-*mvenus*-FLP-*kan*-FLP	This study

**Table 4.** Primer list used in this study.

PA-Cre plasmid construction		
Name	Sequence	Usage
pLVK_pTVK_F	CAGGCATCAAATAAAACGAAAGGCTCAGTC	For constructing pKK1 and pKK2 plasmids using ExoIII cloning (Li and Evans, 1997). Templates: pLVK3 (pLVK_pTVK_F & pLVK3_pTVK3_R), pCMV-rfp-pmag-creC (L3_TagRFP_F & Crec_Term_R), and pCMV-rfp-nmag-creN (L3_TagRFP_F & nMagCreN-R)
pLVK3_pTVK3_R	GGTACCTTTCTCCTCTTTAATGTTTTCGG	
L3_TagRFP_F	GGACGCACTGACCGAAAACATTAAAGAG GAGAAAGGTACCATGGTGTCTAAGGGCGAAG	
Crec_Term_R	CCAGTCTTTCGACTGAGCCTTTCGTTTT ATTTGATGCCTGTTAGTCCCCATCTTCGAGCAG	
nMagCreN-R	TTCGTTTTATTTGATGCCTGTTAGTTCAGCTTGCACCAGG	
YKp0001	ATGCATACTCTTTATGCCCCCGG	For constructing pYK001 and pYK002 plasmids by removing the *rfp* gene from pKK1 and pKK2.
YKp0002	GGTACCTTTCTCCTCTTTAATG	
YKp0053	AACAGGAAATGGTTCCCTGCTGAACC	For constucting pYK016 plasmid by changing the linker between *pmag* and *creC* on pYK001.
YKp0054	GGTACCTTCTGTTTCGCACTGGAATC	
YKp0022	GTGTAGGCTGGAGCTGCTTCG	For constructing pYK017 plasmid using ExoIII cloning. PCR was conducted with the template pYK002 using YKp0022 & YKp0052, YKp0049 & YKp0056 and YKp0055 & YKp0057 to change the order of *nmag* and *creN*.
YKp0049	AAGAGGAGAAAGGTACCATGACCTCTGATGAAGTCAGG	
YKp0052	CATGGTACCTTTCTCC	
YKp0055	CATACTCTTTATGCCCCCGGTGG	
YKp0056	GCATAAAGAGTATGGGTACCGTTCAGCTTGCACCAG	
YKp0057	CTCCAGCCTACACTTATTCTGTTTCGCACTGGAATC	
YKp0058	TTATTCTGTTTCGCACTGGAATCCC	For constructing pYK018 plasmid using ExoIII cloning. PCR was conducted on the template pYK016 with YKp0059 & YKp0060 and pYK017 with YKp0022 & YKp0058 to combine *pmag-creC* and *creN-nmag*.
YKp0059	GCGAAACAGAATAAAGGAGAAAGGTACCATGCATAC	
YKp0060	GCTCCAGCCTACACTTAGTCCCCATCTTCGAGCAGC	
YKp0006	CAGGCATCAAATAAAACGAAAGGCTCAGTCG	For constructing pYK022 plasmid using ExoIII cloning. PCR was conducted on the template pYK001 with YKp0006 & YKp0052 and pYK018 with YKp0049 & YKp0061 to add a terminator.
YKp0061	TTATTTGATGCCTGTTAGTCCCCATCTTCGAGCAGC	
Floxed *cat* plasmid construction		
Name	Sequence	Usage
YKp0026	CGTTTTATTTGATGCCTGATAACTTCGTATAGCATAC	For constructing pYK006 plasmid. Template: pCMV_lox2272_loxp_inverse-*mvenus*_lox2272_loxp.
YKp0027	CATTAAAGAGGAGAAAGGTACCATAACTTCGTATAGGATAC	
YKp0029	CTTATTAGAATTCGCCGCCATGGTGAGCAAGGGCGAG	For constructing pYK007 plasmid using ExoIII cloning. PCR was conducted on the template pTmCherryK3 with YKp0029 & YKp0030 and pYK006 with YKp0031 & YKp0032 to convert *mvenus* to *mcherry*.
YKp0030	CATTATACGAAGTTATCTCGAGTTATCCACGCGTGAGC	
YKp0031	CTCGAGATAACTTCGTATAATG	
YKp0032	GGCGGCGAATTCTAATAAGG	
YKp0035	ATAACTTCGTATAAAGTATCCTATACGAAGTTATGGTACC	For constructing pYK008 plasmid. PCR was conducted on the template pYK007 to delete spacer sequence.
YKp0036	ATGGTGAGCAAGGGCGAGGAGGATAACATGGCCATCATC	
YKp0037	TAACTCGAGATAACTTCGTATAATG	For constructing pYK009 plasmid with the template pYK008 to delete SacI and MluI restriction sites.
YKp0038	CTTGTACAGCTCGTCCATGC	
YKp0039	GAAAAAAATAACTTCGTATAGGATAC	For constructing pYK010 plasmid with the template pYK009 to change the ribosomal binding site (RBS).
YKp0040	CTCCTCTTTAATGTTTTCGGTCAGTGCG	
YKp0041	GAAGTTATAGGAGGAAAAAAATGGTGAGCAAGGGCGAGGAGG	For constructing pYK011 plasmid with the template pYK010 to transfer RBS in floxed site.
YKp0042	GTATAAAGTATCCTATACGAAGTTATCTTTAATGTTTTCGGTCAGTGCG	
YKp0069	CCGAAAACATTAAAGATAACTTCGTATAGCATACATTATACG	For constructing pYK023 with the template pYK011 (YKp0041 & YKp0069) to change lox2272 to loxP.
YKp0083	ATACATTATACGAAGTTATCAGGCATCAAATAAAACG	For constructing pYK028 plasmid with the template pYK023 to change the direction of loxP site.
YKp0084	GCTATACGAAGTTATCTCGAGTTACTTGTACAGCTC	
YKp0072	CGAGCTGTACAAGGAGCTCGAGAAAAAAATCACTGG	For constructing pYK029 plasmid using ExoIII cloning. PCR was conducted on the template pYK028 (YKp0072 YKp0089) and pTVCK4 (YKp0075 & YKp0083) to insert *cat* gene.
YKp0075	CTTGTACAGCTCGTCCATGCCGCCGGTGG	
YKp0089	CGTATAATGTATGCTATACGAAGTTATCTCGAGTTATCCAC	
YKp0109	GAGAAAAAAATCACTGG	For constructing pYK035 plasmid with the template pYK029 to remove *mcherry* gene.
YKp0110	CATTTTTTTCCTCCTATAAC	
Genome integration by λ-Red recombination		
Name	Sequence	Usage
intC_PtetO1_F3	AGTTGTTAAGGTCGCTCACTCCACCTTC TCATCAAGCCAGTCCGCCCATCCCTATCAGTGATAGAGATTG	For genome integration of floxed *mcherry-cat*-FRT-*kan*-FRT or *cat*-FRT-*kan*-FRT at *intC* site by λ-Red recombination. PCR was conducted on the template pYK029 or pYK035.
intC_R	CCGTAGATTTACAGTTCGTCATGGTTCG CTTCAGATCGTTGACAGCCGCAATTCCGGGGATCCGTCGACC	
oMU94	ACCTGCGTGAGCGTACTGGTAAGGCTGC TCGTATCAAAGAGCGTCTTAACGTGAGCAAGGGCGAGGA	For integrating *mcherry*-FRT-*kan*-FRT fragment at *rplS* site. Template: pMU1.
oMU107	GCCAGCCAATTGGCCAGCCCTTCTTAAC AGGATGTCGCTTAAGCGAAATCTTGTGTAGGCTGGAGCTGCT	
oMU92	GTTCTCAGGATCTGGCTTCCCAGGCGG AAGAAAGCTTCGTAGAAGCTGAGGTGAGCAAGGGCGAGGA	For integrating *mvenus*-FRT-*kan*-FRT fragment at *rpsB* site. Template: pMU2.
oMU108	TTGCCGCCTTTCTGCAACTCGAACTATT TTGGGGGAGTTATCAAGCCTTATTGTGTAGGCTGGAGCTGCT	
Primers for sequence check		
Name	Sequence	Usage
M13F	CAGGAAACAGCTATGAC	Sequence check for the pMW118 derivative plasmids.
pM1_pVT_seq_primer1	GGCACCCCAGGCTTTAC	
intC_check_F	GATCGATACTTGCTGTGGTTGATG	Sequence check for the *intC* site.
intC_check_R2	CCTCTTAGTTAAATGGATATAACGAGCCCC	
YKp0077	CACCGTTGATATATCCCAATGGC	Sequence check for the *cat* gene.
YKp0115	CACTCATCGCAGTACTGTTG	
oMU96	TCCAGACTCACTCTCCGGTAGT	Sequence check for the *rplS* site. For the BW25113 derivative strains oMU96 & oMU97; For the W3110 derivative strains oMU96 & oMU110
oMU97	ATAGCCAGTAACAAGACCGCCC	
oMU110	GACAAATTCCACGCAGCAATCTCAC	
oMU26	AAGCAAACAACCTGGGTATTCCGGT	Sequence check for the *rpsB* site
oMU28	CTCGCTCATCCCGGTCACTTACTGA	

When constructing the resistance-gene-deleted strains (YK0085 and YK0138), YK0083 and YK0136 cells were pre-cultivated overnight in LB broth containing 50 µg/mL of ampicillin (Amp, Wako). 10 µL of overnight cultures were inoculated into 2 mL of the M9 medium containing 50 µg/mL of Amp and 0.1 mM isopropyl-β-D-thiogalactopyranoside (IPTG, Wako) in test tubes. The cell cultures were cultivated at 37 °C with shaking. After a 3-hr cultivation period for inducing the expression of PA-Cre system by IPTG, the batch cultures were illuminated by LED blue light (CCS) for 24 hr. The light intensity was adjusted to 6.8 mW, which is equivalent to the intensity in the microscopy experiments. The cell cultures were streaked on LB agar containing 100 µg/mL of Amp. The plates were incubated at 37 °C overnight. After overnight incubation, several colonies were selected. The deletion of the intended gene in these colonies was examined via PCR. The colonies with the intended gene deletion were cultured in LB medium containing 50 µg/mL of Amp at 37 °C. These cell cultures were stored at –80 °C as glycerol stocks.

The genomes of the constructed strains and plasmids were purified using the Promega Genomic DNA Purification kit and the Promega Plasmid Miniprep System, respectively, and the DNA sequence of the modified locus was amplified by Prime STAR (Takara). The sequence was examined by Sanger sequencing using a commercial service (FASMAC).

In microscopy experiments, we used M9 minimal medium, which consisted of M9 salt (Difco), 2 mM MgSO_4_ (Wako), 0.1 mM CaCl_2_ (Wako), 0.2% (w/v) glucose (Wako), and 0.2%(v/v) MEM amino acid (50 x) solution (Sigma). When necessary, antibiotics and chemicals were added at the following final concentrations unless otherwise noted: Amp 50 µg/mL, Km 20 µg/mL, chloramphenicol (Cp, Wako) 15 µg/mL, and IPTG 0.1 mM.

### Calculation of the fraction of resistance-gene-deleted cells in batch culture

YK0083 cells were pre-cultivated in LB broth containing 50 µg/mL of Amp overnight. 100 µL of the overnight culture was spun at 21,500 ×g for 1 min by a centrifuge (CT15E, himac, Hitachi) and resuspended in 1 mL of the M9 minimal medium. This culture was inoculated in 2 mL of the M9 medium containing 50 µg/mL of Amp and 0.1 mM IPTG (except for the ‘no IPTG’ condition) in test tubes covered with aluminum foil for light protection. The starting OD_600_ was adjusted to 0.001, and the cells were cultivated at 37 °C with shaking. After a 3-hr cultivation period for inducing the expression of the PA-Cre system by IPTG, the aluminum foil cover was removed, and the test tubes were exposed to blue light. The light intensity was adjusted to 6.8 mW. The illumination length was determined according to the experimental conditions (Figure 1—figure supplement 2B). For the conditions with an illumination duration of less than 6 hr, the test tubes were again covered with aluminum foil and incubated with shaking so that the total cultivation duration from the start of blue-light illumination was identical (6 hr) across all conditions. Under the ‘no IPTG’ condition, the test tubes were covered with the aluminum foil throughout the incubation period and were not exposed to blue light.

For calculating the fractions of resistance-gene-deleted cells, the cultures exposed to blue-light were diluted to OD_600_ = $1.0\times 10^{-6}$, and 150 µL of the diluted cultures was spread on LB agar containing 100 µg/mL of Amp. The plates were incubated at 37 °C for 18 hr. After incubation, the number of colonies was counted under ambient light or excitation light for examining mCherry fluorescence using stereomicroscope (stereomicroscope: Olympus SZ61; LED source: NIGHTSEA SFA-GR). The fraction of resistance-gene-deleted cells was calculated as the number of non-fluorescent colonies divided by the number of total colonies (Figure 1—figure supplement 2B). To validate this classification, we conducted colony PCR and checked whether the designed deletion was introduced (Figure 1—figure supplement 2C).

We also performed the resistance-gene-deletion experiments under the Cp exposure (Figure 3—figure supplement 2A). YK0083 cells were pre-cultivated in LB broth containing 50 µg/mL of Amp overnight. 100 µL of the overnight culture was spun at 21,500 ×g for 1 min by a centrifuge and resuspended in 1 mL of the M9 minimal medium. This culture was inoculated in 2 mL of the M9 medium containing 50 µg/mL of Amp, 15 µg/mL of Cp and 0.1 mM IPTG in test tubes covered with aluminum foil for light protection. The starting OD_600_ was adjusted to 0.001, and the cells were cultivated at 37 °C with shaking. After a 3 hr cultivation period for inducing the expression of the PA-Cre system by IPTG, the aluminum foil cover was removed, and the test tubes were exposed to blue light for 30 min. The test tubes were again covered with aluminum foil and incubated with shaking for 2 hr. The cultures exposed to blue-light were diluted to OD_600_ = $1.0\times 10^{-6}$, and 150 µL of the diluted cultures was spread on LB agar containing 100 µg/mL of Amp. The plates were incubated at 37 °C overnight. After incubation, we examined the fluorescence of mCherry under the excitation light. The correspondence between the loss of mCherry fluorescence and the absence of *cat* resistance gene was verified by colony PCR (Figure 3—figure supplement 2A).

### Sample preparation and experimental procedures for competition assay

YK0083 cells were pre-cultivated in LB broth containing 50 µg/mL of Amp overnight. 100 µL of the overnight culture was spun at 21,500 ×g for 1 min by a centrifuge and resuspended in 1 mL of the M9 minimal medium. This culture was inoculated in 2 mL of the M9 medium containing 50 µg/mL of Amp, 15 µg/mL of Cp and 0.1 mM IPTG in test tubes covered with aluminum foil for light protection. The starting OD_600_ was adjusted to 0.001, and the cells were cultivated at 37 °C with shaking. After a 3-hr cultivation period for inducing the expression of the PA-Cre system by IPTG, the aluminum foil cover was removed, and the test tubes were exposed to blue light for 30 min. The test tubes were again covered with aluminum foil and incubated with shaking. The cultivation length after blue light illumination was determined according to the experimental conditions (Figure 3—figure supplement 2B). After the cultivation, the cultures were diluted to OD_600_ = $1.0\times 10^{-6}$, and 100 µL of the diluted cultures was spread on LB agar containing 100 µg/mL of Amp. The plates were incubated at 37 °C for 18 hr. After incubation, the number of colonies was counted under ambient light or excitation light for examining mCherry fluorescence using stereomicroscope. The fraction of resistance-gene-deleted cells was calculated as the number of non-fluorescent colonies divided by the number of total colonies (Figure 3—figure supplement 2B).

### Sample preparation and experimental procedures for MIC tests

Cells stored as a glycerol stock were inoculated in the LB broth (Figure 2 and Figure 5—figure supplement 1) or the M9 medium (Figure 3—figure supplement 3C) containing 50 µg/mL of Amp. The cells were cultured at 37 °C with shaking overnight. 100 µL of overnight culture was centrifuged at 21,500×g for 3 min. The supernatant was discarded, and the cells were resuspended in 1.0 mL of the M9 medium. This culture was diluted to the cell density corresponding to OD_600_ = 0.01 in the M9 medium containing 50 µg/mL of Amp and incubated with shaking for 3–4 hr at 37 °C. The cell culture was again diluted to OD_600_ = 0.001 in fresh M9 media containing different concentrations of Cp (Figure 2, Figure 3—figure supplement 3C and Figure 5—figure supplement 1). After 23 hr of incubation, the OD_600_ of these cultures was measured with a spectrometer (UV-1800, Shimadzu) in Figure 2 and Figure 5—figure supplement 1 or with a multi-mode microplate reader (FilterMax F5, Molecular Devices) in Figure 3—figure supplement 3C.

### Microfabrication of mother machine microfluidic device

We created two chromium photomasks, one for the main trench and the other for the observation channels, by laser drawing (DDB-201-TW, Neoark) on mask blanks (CBL4006Du-AZP, Clean Surface Technology). The photoresist on mask blanks was developed in NMD-3 (Tokyo Ohka Kogyo), and the uncovered parts of the chromium layer were removed by MPM-E30 (DNP Fine Chemicals). The remaining photoresist was removed by acetone. The photomasks were rinsed in MilliQ water and air-dried.

We created the mold for the mother machine on a silicon wafer (ID447, ϕ=76.2 mm, University Wafer). First, we spin-coated SU8-2 (MicroChem) on the wafer with the target height of 1.2 µm. The SU8-coated wafer was baked at 65 °C for 1 min and thereafter at 95 °C for 3 min. The SU8-layer was exposed to UV light thrice (each exposure, 22.4 mW/cm^2^, 1.7 sec) using a mask aligner (MA-20, Mikasa). The photomask for the observation channels was used in this step. The wafer was post-baked at 65 °C for 1 min and 95 °C for 3 min after the exposure and developed with the SU8 developer. The wafer was rinsed with isopropanol (Wako) and air-dried.

Next, we spin-coated SU8-3025 (MicroChem) with the target height of 20 µm. The pre-bake was performed at 65 °C for 3 min and thereafter at 95 °C for 7 min. The SU-8 layer was again exposed to UV light (22.4 mW/cm^2^, 30 s) with the photomask for the main trench. The wafer was post-baked at 65 °C for 3 min and 95 °C for 10 min and developed with the SU8 developer. The wafer was rinsed with isopropanol and air-dried.

The Part A and Part B of polydimethylsiloxane (PDMS) resin (Sylgard 184 Silicone Elastomer Kit, Dow Corning) were mixed at the ratio of 10:1 and poured onto the SU-8 mold placed in a tray made of aluminum foil. After removing the air bubbles under decreased pressure, the PDMS was cured at 65 °C for 1 hr. We cut out a PDMS block containing the channel structures and punched holes at both ends of the main trench. The PDMS block was washed with isopropanol and heated at 65 °C for 30 min.

We washed the coverslips (thickness: 0.13–0.17 mm, 24 × 60 mm, Matsunami) by sonication with 10-fold-diluted Contaminon solution (ContaminonR⃝ LS-II, Wako) for 30 min, with 99.5% ethanol (Wako) for 15 min, and with 0.8 M NaOH solution (10-fold diluted 8 M NaOH (Wako)) for 30 min. The coverslips were rinsed with milliQ water by sonication after each washing step. The coverslips were dried at 140 °C for 1 hr.

We exposed the PDMS block and the coverslips to oxygen plasma using a compact etcher (FA-1, SAMCO) and bonded them together at 65 °C for 5 min. After bonding, we inserted the silicone tubes (inner diameter: 1 mm, outer diameter: 2 mm, Tigers Polymer Corporation) into the holes and smeared a small amount of pre-cured PDMS. We cured the PDMS at 65 °C overnight to fix the tubes into the holes tightly.

### Sample preparation and experimental procedures for single-cell time-lapse experiments

Cells stored as a glycerol stock were inoculated in the LB broth containing 50 µg/mL of Amp and cultured at 37 °C overnight with shaking (200 rpm). 200 µL of overnight culture was centrifuged at 21,500×g for 3 min. The supernatant was discarded, and the cells were resuspended in 1.5 mL of the M9 medium. This culture was diluted to OD_600_ = 0.01 in the M9 medium containing 50 µg/mL of Amp, and incubated with shaking for 5–6 hr at 37 °C. The cell culture was again spun at 2350×g for 5 min using a centrifuge (CT6E, himac, Hitachi), and the cells were resuspended in 200 µL of the M9 medium.

Before loading the cells, the growth channels and the main trench in the mother machine device were washed by flowing 0.5 mL of 99.5% ethanol, M9 medium, and 1% (w/v) bovine serum albumin (BSA, Wako) solution sequentially using a syringe. After washing, we introduced the cell suspension into the device and let the cells enter the growth channels by placing the device at 37 °C for 1–2 hr without flow in a dark room. After confirming that the cells were trapped in many growth channels, we started flowing the M9 medium containing 0.1 mM IPTG and 0.1% (w/v) BSA at 2 mL/hr. For experiments with continuous Cp exposure, we also added 15 µg/mL or 30 µg/mL of Cp to the flowing medium from the beginning. We initiated time-lapse image acquisitions after overnight cultivation in the mother machine. We illuminated blue light for 30 min at pre-determined timings in all single-cell time-lapse experiments. The medium flow rate was maintained at 2 mL/hr and increased to 5 mL/hr for 30 min once or twice a day to prevent the formation of cell aggregates in the main trench. In Cp removal experiment, the flowing medium was switched to M9 medium containing 0.1 mM IPTG and 0.1% (w/v) BSA at 2 mL/h 72 hr after blue light exposure stopped.

### Time-lapse image acquisitions and analysis

Time-lapse image acquisitions were performed with ECLIPSE Ti fluorescent microscope (Nikon) equipped with 100× oil immersion objective lens (Plan Apo λ, NA 1.45, Nikon), digital CMOS camera (ORCA-frash, Hamamatsu Photonics), and LED light source (DC2100, Thorlabs) for fluorescence excitation. For acquiring phase-contrast images, the transmitted light was illuminated for 20 ms during *mcherry-cat* gene deletion experiments and for 50 ms during experiments performed with ribosome reporter strains through neutral density and red filters to avoid unintended activation of PA-Cre. Fluorescence images were acquired with appropriate filter cubes (YFP HQ (Nikon) for mVenus and Texas Red (Nikon) for mCherry). In the *mcherry-cat* gene deletion experiments, mCherry fluorescence was obtained every 2 min with excitation of 500 ms. The mCherry and mVenus fluorescence images were acquired every 10 min with the excitation of 100 ms in the experiments with ribosome reporter strains. IPTG was removed from the flowing media after blue-light illumination to avoid unintended gene deletion.

Blue-light illumination on the microscope for gene deletion experiments was regulated by an independent controller. We used a tape LED (7.2 mW, wave length:464∼474 nm, 60 LED/1 m, LED PARADISE) as the blue-light source and surrounded the mother machine device on the microscope stage with this tape LED. The LED light was switched on and off with a timer (REVEX).

Image analysis was performed using ImageJ Fiji (http://fiji.sc/). Image registration was performed by HyperStackReg described in MoMA Macro (Kaiser et al., 2018). Cell segmentation was semi-automated and retouched manually using iPad Pro Sidecar. Semi-automated segmentation and tracking Macro reported previously (Hashimoto et al., 2016) were used in this study. The data from image analysis were further analyzed using Python 3 (https://www.python.org/) with some general packages, including NumPy, SciPy, pandas, Matplotlib, seaborn, FastDTW, and JupyterLab.

The transitions of mCherry-CAT fluorescence intensities and elongation rates in single-cell lineages were classified using hierarchical clustering in Figure 5—figure supplement 2. The full-length single-cell lineage data were used in this analysis. The similarity of each pair of time series was measured by dynamic time warping (Salvador and Chan, 2007). Hierarchical clustering is agglomerative; the averaged dynamic time warping was used when similarity to another time series or cluster was evaluated.

### Cell sampling from the mother machine and whole-genome sequencing

After time-lapse observations performed with the mother machine, we switched the flowing media to the M9 medium containing 0.1% (w/v) BSA and no Cp. We recovered the growth of resistance-gene-deleted cell lineages by culturing them without Cp for more than 6 hr. Subsequently, we collected the media flowing out from the mother machine in a 1.5 mL tube. The OD_600_ of the collected cell suspension was measured with a spectrometer (UV-1800, Shimadzu). The cell suspension was diluted serially with the M9 medium to obtain a cell density corresponding to ${OD}_{600}=3.3\times 10^{-9}$. A total of 200 µL of diluted cell suspension was taken in each well of 96-well plates. The number of cells in the 200 µL of cell suspension k is expected to follow a Poisson distribution $P\,\left(k\right)=\lambda^{k}\,e^{-\lambda }/k!$ with the mean λ = 0.54. Therefore, P⁢(0)=0.58, P⁢(1)=0.31, P⁢(2)=0.085, and P⁢(k≥3)=0.018. The 96-well plates were incubated with shaking at 37 °C for two nights. We performed PCR on samples with high turbidity in wells to confirm the presence or absence of the *cat* gene. We selected both *cat*-positive and *cat*-negative cellular populations from the 96-well plates and cultured them in test tubes overnight at 37 °C. We stored these samples at –80 °C for further analyses.

For the measurement of mCherry-CAT fluorescence intensities of the selected samples (Figure 3—figure supplement 3), cells stored as a glycerol stock were inoculated in the M9 medium containing 50 µg/mL of Amp and cultured at 37 °C with shaking (200 rpm) overnight. 10 µL of overnight culture was inoculated in the M9 medium containing 50 µg/mL of Amp and incubated with shaking for 4 hr at 37 °C. A total of 0.3 µL of cell culture was placed on an agar pad prepared using the M9 medium and 1.5% (w/v) agar (Wako) and covered with a coverslip. Image acquisitions of the samples were performed with ECLIPSE Ti fluorescent microscope (Nikon) equipped with 100× oil immersion objective lens (Plan Apo λ, NA 1.45, Nikon), digital CCD camera (ORCA-R2, Hamamatsu Photonics), and LED light source (DC2100, Thorlabs) for fluorescence excitation. For acquiring phase-contrast images, the transmitted light was illuminated for 50 ms through neutral density. The mCherry fluorescence images were acquired with Texas Red filter cubes and an exposure time of 500 ms.

For the measurement of whole-genome sequencing of the selected samples, cells stored as a glycerol stock were inoculated in 5 mL of the M9 medium and cultured at 37 °C with shaking (200 rpm) overnight. The OD_600_ of overnight culture was measured with a spectrometer. Rifanpicin (Wako) was added to the overnight culture to the final concentration of 300 µg/mL. After 3 hr of incubation, the genomes of these samples were extracted with DNeasy Blood and Tissue kit (QIAGEN). TruSeq DNA PCR Free kit (Illumina) was used for the whole-genome sequencing library preparation. These libraries were sequenced using NovaSeq (Illumina). Library preparation and sequencing were outsourced to Macrogen Japan (Tokyo, Japan). The sequence data were analyzed with breseq (Deatherage and Barrick, 2014).

We evaluated the probability that all of the five cell populations for the whole-genome sequencing were derived from growth-halted cell lineages were 5.5%. This value was obtained as ${\left(\frac{\left(1-0.373\right)\times 0.693}{0.373\times 0.913+\left(1-0.373\right)\times 0.693}\right)}^{5}=0.055$, considering the fraction of growth-restored cell lineages among the resistance-gene-deleted cell lineages (37.3%) and the proportions of the cell lienages that recovered fast growth after Cp removal (91.3% for growth-restored cell lineages; 69.3% for growth-halted cell lineages).

### Data availability

All the data obtained in this study except for high-throughput sequencing data have been deposited to a Github repository (https://github.com/YKogane/History-Dependent-Physiological-Adaptation-2021, copy archived at swh:1:rev:ddbd24c49b29ade0f667a739c3f04b5d66e12488; Koganezawa, 2022). High-throughput sequencing data have been deposited in the NCBI Sequence Read Archive (SRA) under BioProject accession in no. PRJNA774496.

### Code availability

All the codes used for the data analysis are available from a Github repository (https://github.com/YKogane/History-Dependent-Physiological-Adaptation-2021).

## Data Availability

All the data obtained in this study except for high-throughput sequencing data have been deposited to a Github repository (https://github.com/YKogane/History-Dependent-Physiological-Adaptation-2021, copy archived at swh:1:rev:ddbd24c49b29ade0f667a739c3f04b5d66e12488). High-throughput sequencing data have been deposited in the NCBI Sequence Read Archive (SRA) under BioProject accession in no. PRJNA774496. The following datasets were generated: KoganezawaY UmetaniM SatoM WakamotoY 2021History-Dependent-Physiological-Adaptation-2021GitHubHistory-Dependent-Physiological-Adaptation-202110.7554/eLife.74486PMC909033335535492 KoganezawaY UmetaniM SatoM WakamotoY 2021Whole-genome sequence of *Escherichia coli* acquired from Mother MachineNCBI BioProjectPRJNA774496

## Additional files

Transparent reporting form
